# Mitochondrial AAA^+^ protease activity uncovers differential sensitivity of *Drosophila* blood cell lineages to systemic cues

**DOI:** 10.3389/fcell.2025.1606805

**Published:** 2025-11-24

**Authors:** Rajarshi Batabyal, Aman Sharma, Maneesha S. Inamdar

**Affiliations:** 1 Molecular Biology and Genetics Unit, Jawaharlal Nehru Centre for Advanced Scientific Research, Bangalore, India; 2 Molecular Biology and Genetics Unit, Institute for Stem Cell Science and Regenerative Medicine (BRIC-InStem), Bangalore, India

**Keywords:** AAA+ proteases, AFG3L2, YME1L, lymph gland, hematopoiesis, ROS

## Abstract

Hematopoiesis involves balanced blood progenitor proliferation, differentiation, and death, in response to dynamic physiological cues. Previous studies have shown that *Drosophila* lymph gland blood progenitors reside in functionally distinct compartments. Mitochondrial fission-fusion balance and maturity also vary among progenitor compartments and affect cell fate and lineage choice. Here, we show that perturbing mitochondrial homeostasis in *Drosophila* blood cells (hemocytes), can have multiple long-range effects on lymph gland hematopoiesis. Specifically, depletion of the mitochondrial AAA^+^ proteases AFG3L2 or YME1L from the niche or blood progenitors, caused larval lethality. However, depletion from hemocytes alone, gave viable larvae but with a histolysed primary lobe, and an intact and expanded niche. Posterior lobes showed severe hyperproliferation and precocious differentiation. Genetic or pharmacological reduction of ROS restored LG size and differentiation status to control levels, while reducing Hippo pathway activity partially rescued the precocious differentiation. Our study provides the first insights into the roles of mitochondrial AAA^+^ proteases in mediating generation or modulation of systemic signals that control inter-compartmental cross talk between hemocytes and the hematopoietic organ. We propose that mitochondrial homeostasis in hemocytes is a key point of control that helps restrict progenitor differentiation. Given the conservation in AAA^+^ protease functions and in signaling pathways that control hematopoiesis, our studies will help gain insight into systemic control of mammalian hematopoiesis.

## Introduction

Hematopoiesis is controlled by multiple cell-extrinsic and cell-intrinsic mechanisms including transcriptional regulation, signaling pathways, and organelle homeostasis (Mochizuki-Kashio et al., 2021; Nakamura-Ishizu et al., 2014). In *Drosophila*, definitive hematopoiesis initiates at embryonic stage 16 with the emergence of primary lobes of the lymph gland (LG) that are seeded along the anteroposterior axis of the dorsally located cardiac tube. Posterior lobes arise during the later stages of larval development and are major reservoir/reserve pool of progenitors (Banerjee et al., 2019). During pupariation, the LG progenitors differentiate into hemocytes and dissociate into circulation. Previous studies showed functional anteroposterior compartmentalization of the larval lymph gland (Rodrigues et al., 2021a). Further, progenitors show localized immune response to specific systemic cues such as bacterial infection or wasp parasitism. This suggests different signal sensing or regulatory mechanisms may operate in progenitor subsets.

**FIGURE 1:**
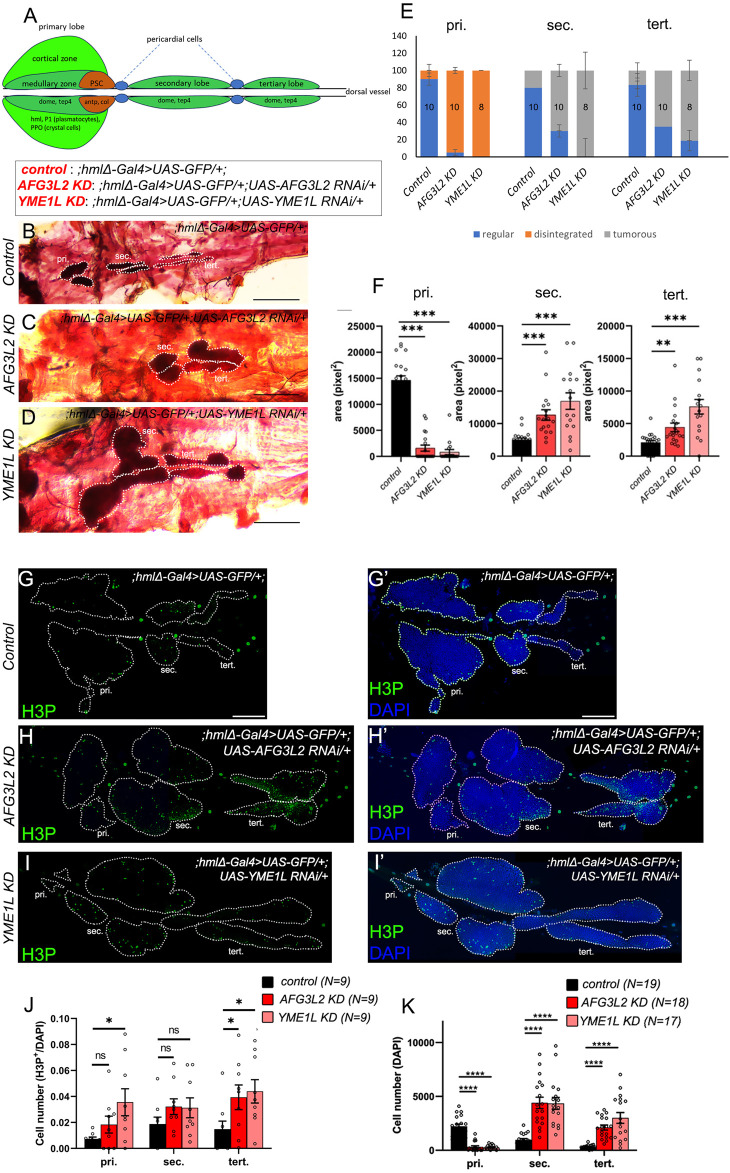
Hemocyte-specific AAA^+^ protease depletion leads to disintegration of primary lobes and hyperplasia of posterior lobes of lymph glands. **(A)** Schematic representation of the expression of indicated markers in different populations of progenitors, differentiated hemocytes and the PSC (posterior signalling centre) in the wandering third instar lymph gland. **(B–D)** Neutral red staining of *control* (;*hmlΔ-Gal4>UAS-GFP/+;)* **(B)**, *AFG3L2 KD (;hmlΔ-Gal4>UAS-GFP/+;UAS-AFG3L2 RNAi/+)* **(C)** and *YME1L KD (;hmlΔ-Gal4>UAS-GFP/+;UAS-YME1L RNAi/+)* **(D)** lymph glands. Dotted lines mark the lobes of the lymph gland. **(E)** Quantification of morphology of the lymph gland lobes in *control* (;*hmlΔ-Gal4>UAS-GFP/+;)*, *AFG3L2 KD (;hmlΔ-Gal4>UAS-GFP/+;UAS-AFG3L2 RNAi/+)* and *YME1L KD (;hmlΔ-Gal4>UAS-GFP/+;UAS-YME1L RNAi/+)*, (number of samples imaged of each genotype mentioned in the bars). **(F)** Quantification of area in pixel^2^ of primary, secondary and tertiary lobes of *control* (;*hmlΔ-Gal4>UAS-GFP/+;)*, *AFG3L2 KD (;hmlΔ-Gal4>UAS-GFP/+;UAS-AFG3L2 RNAi/+)* and *YME1L KD (;hmlΔ-Gal4>UAS-GFP/+;UAS-YME1L RNAi/+)*, **(G–I’)** Lymph gland lobes of *control* (;*hmlΔ-Gal4>UAS-GFP/+;)*, *AFG3L2 KD (;hmlΔ-Gal4>UAS-GFP/+;UAS-AFG3L2 RNAi/+)* and *YME1L KD (;hmlΔ-Gal4>UAS-GFP/+;UAS-YME1L RNAi/+)* marked with H3P^+^ (green) proliferating cells **(G,H,I)** and DAPI^+^ (blue) cells **(G’,H’,I’)**. **(J,K)** Quantification of H3P^+^/DAPI proportion **(J)** and DAPI^+^ **(K)** cell numbers across the different lobes of control (;*hmlΔ-Gal4>UAS-GFP/+;)*, *AFG3L2 KD (;hmlΔ-Gal4>UAS-GFP/+;UAS-AFG3L2 RNAi/+)* and *YME1L KD (;hmlΔ-Gal4>UAS-GFP/+;UAS-YME1L RNAi/+)*, lymph glands. Number of samples quantified for each genotype mentioned above the graphs in colour coded labels. Images represented are maximum intensity projections of individual slices. White dotted lines are used to mark the lymph gland lobes (pri, primary lobes, sec, secondary lobes, tert, tertiary lobes). Error bars represent SEM. Statistical significance was estimated using Students’ t-test with Welch’s correction (ns–nonsignificant, *p < 0.05, **p < 0.01, ***p < 0.001, ****p < 0.0001). Scale bars in **(A–C)** – 200 μm, and in **(E–G’)** - 100 µm.

The LG primary lobes have a signaling niche (posterior signaling center or PSC) that maintains quiescent progenitors and prohemocytes in the medullary zone (MZ), via fibroblast growth factor (FGF) and Hedgehog signalling (Dey et al., 2016; Mandal et al., 2007; Mondal et al., 2011). The outermost cortical zone (CZ) harbors differentiated hemocytes. A schematic describing the various zones of the larval lymph gland and their associated cellular markers are mentioned in Figure 1A. Larval hemocytes function in immunity, development, and signaling crosstalk with other tissues. A majority of the hemocytes in the CZ and circulation are plasmatocytes (∼90%), responsible for phagocytic activities during development and bacterial infections. Crystal cells are less frequent (<10%) and function in melanization and wound healing. Lamellocytes emerge during immune challenges such as wasp infestation (Banerjee et al., 2019; Letourneau et al., 2016).

Extensive studies on LG primary lobes revealed conserved mechanisms regulating hematopoiesis. Further, compared to primary lobe progenitors, their posterior lobe counterparts respond differentially to systemic cues and are developmentally distinct (Rodrigues et al., 2021a). Additionally, posterior lobe progenitors have lower mitochondrial content, indicating their immaturity compared to primary lobe progenitors, supporting recently established concepts of heterogeneity among progenitors across LG lobes (Ray et al., 2021).

Mitochondrial homeostasis attributes such as morphology, metabolism, ROS (reactive oxygen species) and calcium levels regulate HSC (hematopoietic stem cells) quiescence, lineage bias, and progenitor differentiation in both vertebrates and invertebrates (Ito and Ito, 2018; Myers et al., 2018). Hence, perturbations in conserved genes essential for mitochondrial health lead to severe pathophysiological conditions including hematological malignancies (Snoeck, 2017; Myers et al., 2018). We have recently shown that the key regulators of mitochondrial fission and fusion, Drp1 and Marf respectively, affect *Drosophila* blood progenitor differentiation, in association with the conserved regulator of hematopoiesis, Asrij/OCIAD1 (Ray et al., 2021). Furthermore, high throughput interactome studies revealed that OCIAD1 interacts with other critical mitochondrial homeostasis regulators, including the mitochondrial AAA^+^ proteases.

The conserved AAA (ATPases Associated with diverse cellular Activities)-domain-containing family of proteases localizing to the mitochondria, perform diverse roles during both normal development and diseased conditions (Leonhard et al., 1996; Opalińska and Jańska, 2018; Patron et al., 2018). Three mitochondrial AAA^+^ proteases form two distinct protease complexes in the inner mitochondrial membrane (IMM), where they degrade misfolded/unfolded protein aggregates or cleave other proteins to regulate their functions. The m-AAA protease complex, comprising homohexamers of AFG3L2 (ATPase family gene 3-like 2) or heterohexamers of AFG3L2 and Paraplegin (SPG7), exposes its catalytic domains to the matrix. Conversely, the i-AAA protease complex, consisting solely of YME1L/YME1L1 (yeast mitochondrial escape 1 like 1) subunits, exposes its catalytic domains to the Intermembrane space (IMS) between the outer mitochondrial membrane (OMM) and inner mitochondrial membrane (IMM). As these proteases exert their activities on both sides of the IMM, they control the proteome composition and protein quality of the IMS, matrix, and the IMM, resulting in the regulation of multiple aspects of mitochondrial homeostasis. (Leonhard et al., 1996; Opalińska and Jańska, 2018). For example, depletion of both the proteases have been shown to negatively affect mitochondrial morphology through a combinatorial mechanism involving conserved mitochondrial morphology regulators like OMA1 and OPA1 (Anand et al., 2014; Consolato et al., 2018). Depletion of these proteases also lead to accumulation of misfolded electron transport chain complex proteins in the IMM, leading to ROS generation and oxidative stress (Qi et al., 2016; Pareek and Pallanck, 2020). Apart from the aforementioned mitochondrial homeostasis aspects the proteases have been directly linked to regulating mitophagy, mtDNA organization, mitochondrial calcium levels in various contexts including neurodegeneration and normal neuronal development in diverse model systems including *Drosophila*, mice and cell culture (Qi et al., 2016; Kondadi et al., 2014; König et al., 2016; Li et al., 2016; Wani et al., 2022).

Due to the significant conservation of AAA^+^ proteases as a family of proteins and the conserved roles of OCIAD1 in hematopoiesis regulation, we hypothesized that their interaction with OCIAD1 would imply a functional role for the proteases in hematopoiesis. However, given the critical roles that these proteases play in multiple aspects of mitochondrial homeostasis, parsing their additional roles is difficult as any perturbations lead to major effects. In this context the *Drosophila* hematopoietic system offers a powerful model to dissect developmental and lineage-specific roles, as blood cells (hemocytes) have innate immune functions but do not carry out oxygen exchange.

Here, we show that mitochondrial AAA^+^ proteases AFG3L2 and YME1L (referred to as AAA^+^ proteases henceforth) are critical for long range regulation of hematopoietic homeostasis through their control of mitochondrial ROS and downstream signaling.

## Results

### Mitochondrial AAA^+^ protease depletion from blood progenitors leads to larval lethality

We first validated that OCIAD1 interacts with the mitochondrial AAA^+^ proteases, by co-immunoprecipitation experiments on the human immortalized cell line HEK293T (Supplementary Figure S1A). To investigate the relevance of AAA^+^ proteases in hematopoiesis, we surveyed the Leukemia MILE (Microarray Innovations in Leukemia) datasets available in the BloodSpot database (https://www.fobinf.com/?gene=AFG3L2&dataset=all_mile;https://www.fobinf.com/?gene=YME1L1&dataset=all_mile) and found that both AFG3L2 and YME1L have elevated expressions across various subtypes of leukemia including AML (acute myeloid leukemia), ALL (acute lymphoblastic leukemia) and CML (chronic myelogenous leukemia), when compared to healthy bone marrow (Bagger et al., 2019; Haferlach et al., 2008). Further, HemaExplorer datasets (https://www.fobinf.com/?gene=AFG3L2&dataset=nl_human_data_HemaExp_v_1; https://www.fobinf.com/?gene=YME1L12&dataset=nl_human_data_HemaExp_v_1) showed that AAA^+^ protease expression was highest in the earliest blood stem and progenitor populations of the bone marrow compared to differentiated blood cells such as B cells and T cells (Bagger et al., 2019; Bagger et al., 2013) (Supplementary Figure S1B,C), suggesting AAA^+^ regulation may control hematopoietic homeostasis. Further, upon searching the Fly Hemocyte Atlas (http://big2.hanyang.ac.kr/flyscrna/), a single-cell RNA-sequencing database for *Drosophila* hemocytes (Cho et al., 2020) reveal that the expression of AFG3L2 (CG6512) and YME1L are higher in differentiated hemocytes like plasmatocytes and adipohemocytes compared to progenitors or pre-progenitors (Supplementary Figure S1D). Hence, we used the powerful and relevant model of *Drosophila* larval hematopoiesis, to investigate the possible roles of the proteases in hematopoiesis.

Knockdown of either *afg3l2* or *yme1l* from progenitors [using *dome-Gal4>UAS-GFP* or *domeMeso-Gal4>UAS-GFP* or *tep4-Gal4>UAS-GFP* drivers], or from the niche [using *pCol85-Gal4>UAS-GFP* or *antp-Gal4* drivers] resulted in over 95% lethality on YME1L depletion and about 50% lethality in AFG3L2 depleted larvae by the third instar stage (Supplementary Figures S2A–E). This suggested that AFG3L2 and YME1L function in blood cells is essential for larval health and survival.

### AAA^+^ protease depletion from hemocytes leads to precocious differentiation of progenitors

Blood progenitors are also influenced by the status of differentiated hemocytes and systemic signals. Hemocyte-specific knockdown (KD), using *hmlΔ-Gal4>UAS-GFP* (*AFG3L2 KD*–*hmlΔ-Gal4>UAS-GFP/+;UAS-AFG3L2 RNAi/+* or *YME1L KD* - *hmlΔ-Gal4>UAS-GFP/+;UAS-YME1L RNAi/*+), showed a ∼50% decrease in the pupariation rate of *YME1L KD* flies, suggesting impaired hemocyte function in metamorphosis (Supplementary Figure S2F). While there was no significant change in the eclosion rate of survivors (Supplementary Figure S2G), there was a mild yet significant increase in the life span of *YME1L KD* flies (median survival: *control* – 54 days, *AFG3L2 KD* – 52.5 days, *YME1L KD* – 59 days; Supplementary Figure S2H). To further validate the functional efficacy of the knockdown of the proteases in the hemocytes using *hmlΔ-Gal4>UAS-GFP*, aspects of mitochondrial morphology like mean and median branch lengths were probed in control and AAA + KD backgrounds, which revealed severely impaired mitochondrial morphology in the KD backgrounds (Supplementary Figure S3A–E) as previously described in other model organisms (Anand et al., 2014; Consolato et al., 2018).

To probe for effects of hemocyte-specific depletion of AAA^+^ proteases on larval hematopoiesis, we first examined the gross LG morphology in third instar larvae, by neutral red staining, as previously described (Rodrigues et al., 2021b). Though the *hmlΔ-Gal4* driver is active only in differentiated hemocytes of the CZ and in circulation, we observed effects on all LG lobes, including posterior lobes that harbour only progenitors. Primary lobes were partially or completely absent, while posterior lobes exhibited severe hyperplasia with multiple tumorous structures (Figures 1A–D). We also found that the size of the posterior lobes was significantly higher in the AAA + KD LGs as quantified in Figure 1F. Total cell counts (DAPI^+^ nuclei) were significantly high in *AFG3L2 KD and YME1L KD* posterior lobes (Figures 1G–K. Although the number of H3P+ (mitotically active) proliferating cells were higher in the KD backgrounds, their increase was proportionate with the increase in total cell numbers (Figures 1G–J). Stage-specific analysis during larval development [ 60 h After Egg Laying (AEL) (second instar), 72 h AEL (early third instar), 96 h AEL (mid third instar), and 120 h AEL (late third instar)] showed relatively larger primary lobes in *AAA* ^*+*^ *KD* second instar and early third instar LGs with a concomitant increase in numbers of DAPI^+^ cells and Hml^+^ (Hemolectin) hemocytes. Subsequently, primary lobes histolysed and posterior lobes exhibited over-proliferation by mid third instar stage, accompanied with emergence of Hml^+^ hemocytes in the posterior lobes (Supplementary Figure S4A–D).

In summary, we show that AAA^+^ proteases in *Drosophila* blood progenitors and PSC are essential for survival. In parallel, these proteases regulate hematopoietic homeostasis through their functions in the differentiated hemocytes even though not negatively impacting the life span of *AAA* ^*+*^ *KD* flies. Higher number of H3P^+^ proliferating cells and Hml^+^ cells emerging in the posterior lobes of *AAA* ^*+*^ *KD* third instar LGs indicate proliferation and premature differentiation of the posterior lobe progenitors, which we have previously shown to be more resilient to differentiation than the primary lobe progenitors (Mandal et al., 2007).

### Differential sensitivity of niche and progenitor cells to AAA^+^ protease depletion in differentiated hemocytes

During normal larval development under homeostatic conditions, the LG primary lobe progenitors differentiate to plasmatocytes and crystal cells, while the posterior lobe progenitors remain quiescent. Upon receiving specific cues including signaling pathway modulations and immune challenges, anterior progenitors differentiate into additional plasmatocytes and crystal cells, while lamellocytes are induced only in response to wasp infestation (Banerjee et al., 2019; Rodrigues et al., 2021a).

**FIGURE 2:**
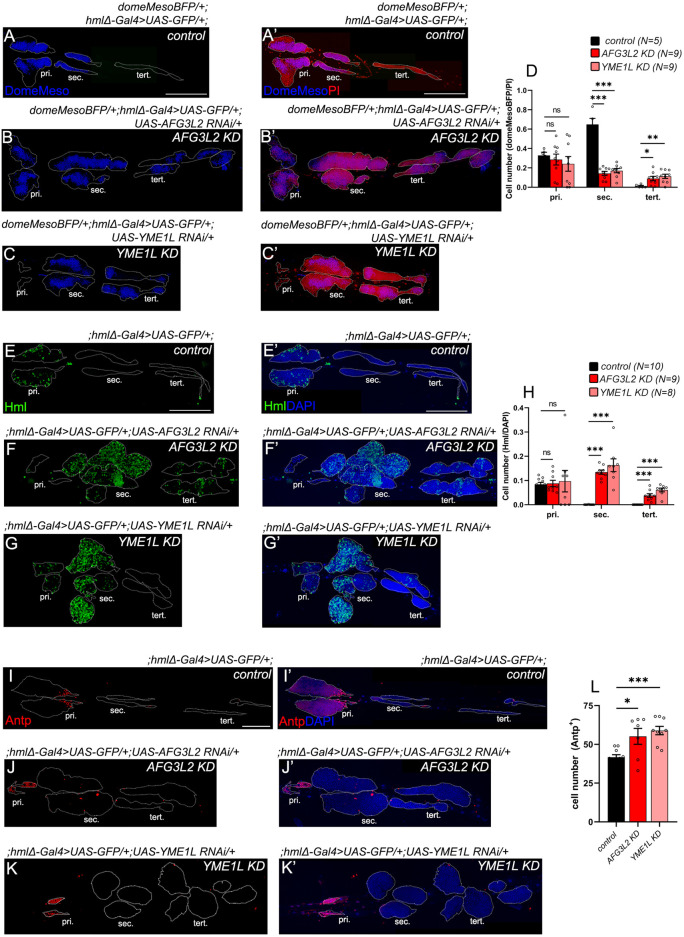
AAA^+^ proteases depletion in hemocytes lead to premature differentiation of the lymph glands. **(A–C’)** Lymph gland lobes of *control (domeMesoBFP/+;hmlΔ-Gal4>UAS-GFP/+;) AFG3L2 KD (domeMesoBFP/+;hmlΔ-Gal4>UAS-GFP/+;UAS-AFG3L2 RNAi/+) and YME1L KD (domeMesoBFP/+;hmlΔ-Gal4>UAS-GFP/+;UAS-YME1L RNAi/+)* genotypes, marked with DomeMesoBFP^+^ (blue) progenitors **(A–C’)**, and PI^+^ (propidium iodide) (red) nuclei **(A’,B’,C’)**. **(D)** Quantification of domeMesoBFP^+^ cell fraction across the lobes *in control, AFG3L2 KD and YME1L KD* lymph glands. **(E–G’)**) Lymph gland lobes of *control* (;*hmlΔ-Gal4>UAS-GFP/+;)*, *AFG3L2 KD (;hmlΔ-Gal4>UAS-GFP/+;UAS-AFG3L2 RNAi/+)* and *YME1L KD (;hmlΔ-Gal4>UAS-GFP/+;UAS-YME1L RNAi/+),* marked with Hml^+^ (green) hemocytes and DAPI^+^ (blue) nuclei **(E’,F’,G’)**. **(H)** Quantification of Hml^+^ cell fraction across the lobes in *control* (;*hmlΔ-Gal4>UAS-GFP/+;)*, *AFG3L2 KD (;hmlΔ-Gal4>UAS-GFP/+;UAS-AFG3L2 RNAi/+)* and *YME1L KD (;hmlΔ-Gal4>UAS-GFP/+;UAS-YME1L RNAi/+)* lymph glands. Images represented are maximum intensity projections of individual slices. White dotted lines are used to mark the lymph gland lobes (pri, primary lobes, sec, secondary lobes, tert, tertiary lobes). Error bars represent SEM. Statistical significance was estimated using Students’ t-test with Welch’s correction (ns–nonsignificant, *p < 0.05, **p < 0.01, ***p < 0.001, ****p < 0.0001). Scale bars – 200 µm. **(I–K’)** Lymph gland lobes of *control* (;*hmlΔ-Gal4>UAS-GFP/+;)*, *AFG3L2 KD (;hmlΔ-Gal4>UAS-GFP/+;UAS-AFG3L2 RNAi/+)* and *YME1L KD (;hmlΔ-Gal4>UAS-GFP/+;UAS-YME1L RNAi/+)* genotypes labelled with PSC marker Antp (red) and nuclear marker DAPI (blue) **(A’,B’,C’)**. (L) Quantification of Antp^+^ cell numbers across the lobes in *control* (;*hmlΔ-Gal4>UAS-GFP/+;) (N =10), AFG3L2 KD (;hmlΔ-Gal4>UAS-GFP/+;UAS-AFG3L2 RNAi/+) (N = 7) and YME1L KD (;hmlΔ-Gal4>UAS-GFP/+;UAS-YME1L RNAi/+) (N = 9)* lymph glands. Images represented are maximum intensity projections of individual slices. White dotted lines are used to mark the lymph gland lobes (pri, primary lobes, sec, secondary lobes, tert, tertiary lobes). Error bars represent SEM.

Remnants of the histolysed primary lobes of *AAA* ^*+*^ *KD* larval LGs showed no significant change in proportion of progenitors or hemocytes from the *control* samples (Figures 2A–H). However, there was a marked decrease in DomeMesoBFP^+^/PI (progenitor/total nuclei) proportions in the secondary lobes with an increase in the tertiary lobes in AAA + KD backgrounds. (Figures 2A–D; *control–domeMesoBFP/+;hmlΔ-Gal4>UAS-GFP/+; AFG3L2 KD - domeMesoBFP/+;hmlΔ-Gal4>UAS-GFP/+;UAS-AFG3L2 RNAi/+, YME1L KD - domeMesoBFP/+;hmlΔ-Gal4>UAS-GFP/+;UAS-YME1L RNAi/+)*. Further, there was a dramatic increase in Hml^+^ hemocyte numbers in *AAA* ^*+*^ *KD* LG posterior lobes (Figures 2E–H), with more drastic effects on the secondary lobes compared to tertiary lobes. There was a minor increase in the Antp^+^ PSC cell numbers as well in the AAA + KD primary lobes (Figures 2I–L). We next examined the differentiation status of the expanded posterior lobes.

### Unbiased differentiation of the AAA^+^ progenitors to plasmatocytes, crystal cells and lamellocytes

**FIGURE 3:**
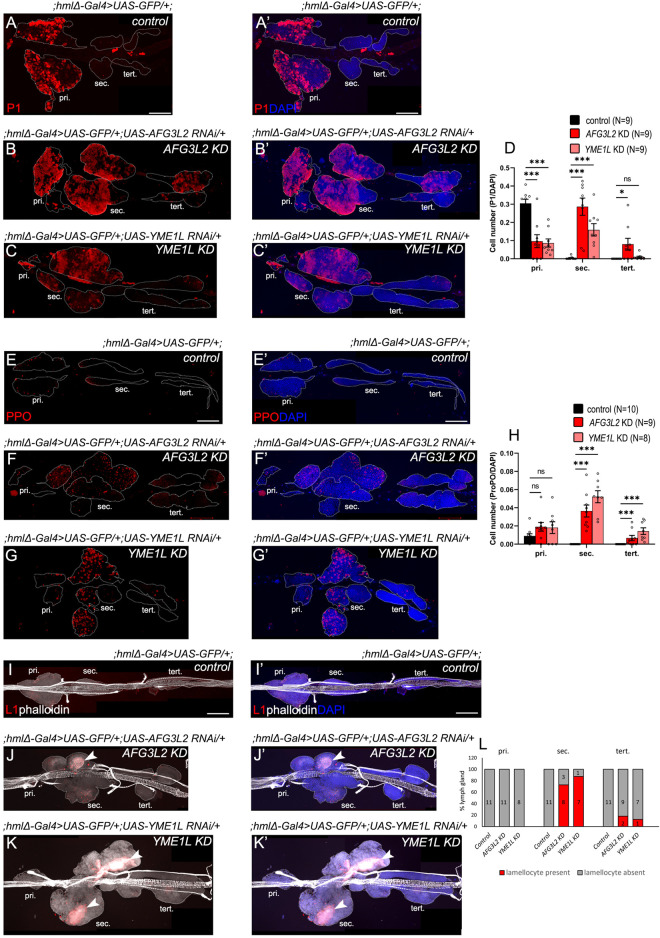
Hemocyte-specific AAA^+^ protease depletion leads to precocious and unbiased differentiation of the progenitors. **(A–C’,E–G’,I–K’)** Lymph gland lobes of *control* (;*hmlΔ-Gal4>UAS-GFP/+;)* **(A,A’,E,E’,I,I’)**, *AFG3L2 KD (;hmlΔ-Gal4>UAS-GFP/+;UAS-AFG3L2 RNAi/+)* **(B,B’,F,F’,J,J’)** and *YME1L KD (;hmlΔ-Gal4>UAS-GFP/+;UAS-YME1L RNAi/+)* **(C,C’,G,G’,K,K’)** genotypes, marked with P1^+^ (red) plasmatocytes **(A–C’)**, PPO^+^ (red) crystal cells **(E–G’)**, and L1^+^ (red) phalloidin^high^ (white) lamellocytes **(I–K’)** (marked with white arrowheads). DAPI (blue) used to mark cells. **(D)** Quantification of P1^+^ cell fraction across the lobes in *control* (;*hmlΔ-Gal4>UAS-GFP/+;)*, *AFG3L2 KD (;hmlΔ-Gal4>UAS-GFP/+;UAS-AFG3L2 RNAi/+)* and *YME1L KD (;hmlΔ-Gal4>UAS-GFP/+;UAS-YME1L RNAi/+)* lymph glands. **(H)** Quantification of absolute PPO^+^ cell numbers across the lobes in *control* (;*hmlΔ-Gal4>UAS-GFP/+;)*, *AFG3L2 KD (;hmlΔ-Gal4>UAS-GFP/+;UAS-AFG3L2 RNAi/+)* and *YME1L KD (;hmlΔ-Gal4>UAS-GFP/+;UAS-YME1L RNAi/+)* lymph glands. **(L)** Quantification of the proportion of lymph gland lobes in which lamellocytes were present in *control* (;*hmlΔ-Gal4>UAS-GFP/+;)*, *AFG3L2 KD (;hmlΔ-Gal4>UAS-GFP/+;UAS-AFG3L2 RNAi/+)* and *YME1L KD (;hmlΔ-Gal4>UAS-GFP/+;UAS-YME1L RNAi/+)* lymph glands. Number of samples quantified for each genotype mentioned above the graphs in colour coded labels or in individual bars as in **(L)**. Images represented are maximum intensity projections of individual slices. White dotted lines are used to mark the lymph gland lobes (pri, primary lobes; sec, secondary lobes; tert, tertiary lobes). Error bars represent SEM. Statistical significance was estimated using Students’ t-test with Welch’s correction (ns–nonsignificant, *p < 0.05, **p < 0.01, ***p < 0.001, ****p < 0.0001). Scale bars–100 µm.

Expectedly, *AAA* ^*+*^ *KD* lymph glands had significantly elevated numbers of P1^+^ plasmatocytes, which are typically Hml^+^, and PPO^+^ (Prophenoloxidase) crystal cells in the posterior lobes, compared to controls (Figures 3A–H). Surprisingly, L1^+^phalloidin^high^ lamellocytes which emerge only in situations such as wasp infestations, were also present in the *AAA* ^*+*^ *KD* secondary lobes (Figures 3I–L, marked by white arrowheads). As observed previously, the differentiation phenotypes were more drastic in the secondary lobes compared to the tertiary lobes (Figures 3D,H,L). In circulation, the proportion of Hml^+^ cells among total DAPI^+^ cells was lower in *AAA* ^*+*^ *KD* samples, likely due to the increase in Hml^−^ or Hml^low^ hemocytes (Supplementary Figure S5A–D). P1+ plasmatocyte numbers were significantly lower in AFG3L2 KD samples, consistent with lower Hml + hemocyte numbers while that of YME1L KD samples were comparable to that of the control samples (Supplementary Figure S5E–H). Further, PPO^+^ crystal cells were increased in circulation in AAA^+^ KD larvae as observed in the *AAA* ^*+*^ *KD* LGs (Supplementary Figure S5I–L). Larger L1^+^phalloidin^high^ lamellocytes were also present among *AAA* ^*+*^ *KD* circulating hemocytes in significant numbers (Supplementary Figure S5M–P). The total number of circulating hemocytes however were unchanged in AFG3L2 KD larvae, and slightly decreased in YME1L KD larvae, indicating that the histolysed primary lobes in the AAA + KD backgrounds do not significantly contribute to the circulating hemocyte pool (Supplementary Figure S5Q). Taken together, our data demonstrate that depleting AAA^+^ proteases in differentiated hemocytes results in unbiased differentiation of the progenitor population in *AAA* ^*+*^ *KD* LGs, leading to increased numbers of all three types of hemocytes.

### AAA^+^ proteases in differentiated hemocytes regulate *Drosophila* hematopoiesis by controlling ROS levels

During hematopoiesis, cellular ROS levels regulate critical cell fate decisions, including differentiation, maintenance of quiescence and lineage bias, through regulation of signaling pathways (Filippi and Ghaffari, 2019; Anderson et al., 2017). As mentioned in the introduction, as both AFG3L2 and YME1L have been shown to regulate ROS levels, we hypothesized that AAA^+^ protease depletion from hemocytes may lead to increased ROS levels and thereby affect pathways involved in blood progenitor maintenance.

**FIGURE 4:**
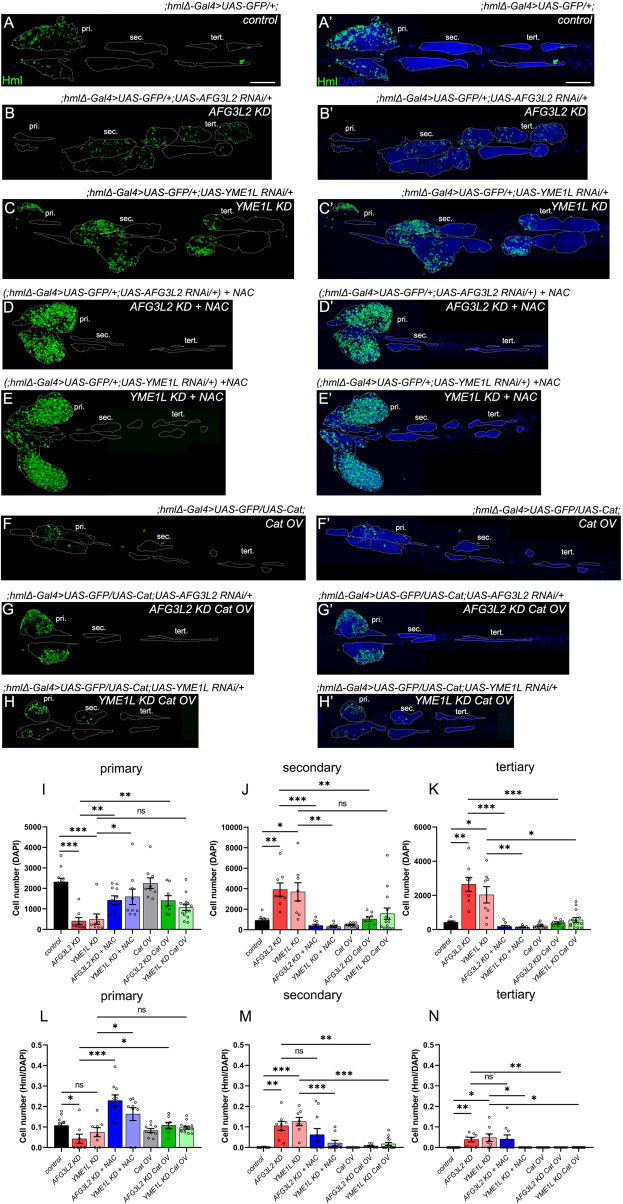
AAA^+^ proteases in differentiated hemocytes regulate *Drosophila* hematopoiesis by controlling ROS levels. **(A–C’)** Lymph gland lobes of *control* (;*hmlΔ-Gal4>UAS-GFP/+;)* **(A,A’)**, *AFG3L2 KD (;hmlΔ-Gal4>UAS-GFP/+;UAS-AFG3L2 RNAi/+)* **(B,B’)** and *YME1L KD (;hmlΔ-Gal4>UAS-GFP/+;UAS-YME1L RNAi/+)* **(C,C’)** genotypes labelled with differentiation marker Hml (green) and nuclear marker DAPI (blue), without any treatment. **(D-E’)** Lymph gland lobes of *AFG3L2 KD (;hmlΔ-Gal4>UAS-GFP/+;UAS-AFG3L2 RNAi/+) and YME1L KD (;hmlΔ-Gal4>UAS-GFP/+;UAS-YME1L RNAi/+)* samples, upon NAC treatment, marked with Hml (green) and DAPI (blue). **(F,F’)** Lymph gland lobes of *Cat OV (;hmlΔ-Gal4>UAS-GFP/UAS-Cat;) samples*, marked with Hml (green) and DAPI (blue). **(G-H’)** Lymph gland lobes of *AFG3L2 KD Cat OV (;hmlΔ-Gal4>UAS-GFP/UAS-Cat;UAS-AFG3L2 RNAi/+)* and *YME1L KD Cat OV (;hmlΔ-Gal4>UAS-GFP/UAS-Cat;UAS-YME1L RNAi/+)* samples, marked with Hml (green) and DAPI (blue). **(I–K)** Quantification of DAPI^+^ cell numbers across the primary **(I)**, secondary **(J)** and tertiary **(K)** lobes of *control* (;*hmlΔ-Gal4>UAS-GFP/+;)* (N – 11), *AFG3L2 KD (;hmlΔ-Gal4>UAS-GFP/+;UAS-AFG3L2 RNAi/+)* (N – 8), *YME1L KD (;hmlΔ-Gal4>UAS-GFP/+;UAS-YME1L RNAi/+)* (N – 8), *AFG3L2 KD (;hmlΔ-Gal4>UAS-GFP/+;UAS-AFG3L2 RNAi/+) + NAC* (N- 10), *YME1L KD (;hmlΔ-Gal4>UAS-GFP/+;UAS-YME1L RNAi/+) + NAC* (N – 9), *Cat OV (;hmlΔ-Gal4>UAS-GFP/UAS-Cat;)* (N-9), *AFG3L2 KD Cat OV (;hmlΔ-Gal4>UAS-GFP/UAS-Cat;UAS-AFG3L2 RNAi/+)* (N-8) and *YME1L KD Cat OV (;hmlΔ-Gal4>UAS-GFP/UAS-Cat;UAS-YME1L RNAi/+)* (N-14) genotypes. **(L–N)** Quantification of Hml^+^/DAPI^+^ cell fraction across the primary **(I)**, secondary **(J)** and tertiary **(K)** lobes of *control* (;*hmlΔ-Gal4>UAS-GFP/+;)* (N – 11), *AFG3L2 KD (;hmlΔ-Gal4>UAS-GFP/+;UAS-AFG3L2 RNAi/+)* (N – 8), *YME1L KD (;hmlΔ-Gal4>UAS-GFP/+;UAS-YME1L RNAi/+)* (N – 8), *AFG3L2 KD (;hmlΔ-Gal4>UAS-GFP/+;UAS-AFG3L2 RNAi/+) + NAC* (N- 10), *YME1L KD (;hmlΔ-Gal4>UAS-GFP/+;UAS-YME1L RNAi/+) + NAC* (N – 9), *Cat OV (;hmlΔ-Gal4>UAS-GFP/UAS-Cat;)* (N-9), *AFG3L2 KD Cat OV (;hmlΔ-Gal4>UAS-GFP/UAS-Cat;UAS-AFG3L2 RNAi/+)* (N-8) and *YME1L KD Cat OV (;hmlΔ-Gal4>UAS-GFP/UAS-Cat;UAS-YME1L RNAi/+)* (N-14) genotypes. Images represented are maximum intensity projections of individual slices. White dotted lines are used to mark the lymph gland lobes (pri, primary lobes; sec, secondary lobes; tert, tertiary lobes). Error bars represent SEM. Statistical significance was estimated using Students’ t-test with Welch’s correction (ns–nonsignificant, *p < 0.05, **p < 0.01, ***p < 0.001, ****p < 0.0001). Scale bars – 100 µm.

Probing for ROS levels in the circulating hemocytes using the cellular ROS sensitive dye, DHE (dihydroethidium), revealed higher DHE intensity in *AAA* ^*+*^ *KD* hemocytes (Supplementary Figure S6A–D). As previously reported, cells with lower Hml intensity had higher DHE intensity (Myers et al., 2018). Since ROS levels in *AAA* ^*+*^ *KD* hemocytes were higher, we explored the possibility of rescuing the *AAA* ^*+*^ *KD* LG phenotypes by reducing ROS levels. *AAA* ^*+*^ *KD* larvae grown in food containing 10 mg/mL NAC (N-acetyl cysteine), (Chen et al., 2023), a free radical scavenger that controls ROS levels by promoting glutathione production (Kelly, 1998), resulted in intact primary lobes in the *AAA* ^*+*^ *KD* larvae *(AFG3L2 KD + NAC and YME1L KD + NAC)* (Figures 4D,E’), although the DAPI^+^ cell numbers were elevated (Figure 4I). Further, there were higher number of Hml^+^ cells in the NAC-treated *AAA* ^*+*^ *KD* LG primary lobes compared to that of untreated *control* (Figures 4A–E,L,M’). This suggests that NAC treatment could rescue *AAA* ^*+*^ *KD* phenotypes of primary lobe disintegration, but not that of differentiation of the resident progenitors. Interestingly, there was complete rescue of the *AAA* ^*+*^ *KD* phenotype in the posterior lobes upon NAC-treatment, with DAPI^+^ cell numbers restored to control levels and absence of Hml^+^ hemocytes (Figures 4A,A’,D–E’).

To validate the specific role of ROS levels on *AAA* ^*+*^ *KD* phenotypes, we overexpressed the ROS scavenger catalase (Cat) in *AAA* ^*+*^ *KD* hemocytes. Compared to the *AAA* ^*+*^ *KD* lymph glands (Figures 4B,C'), *AFG3L2 KD Cat OV* (;*hmlΔ-Gal4>UAS-GFP/UAS-Cat;UAS-AFG3L2 RNAi/+*) and *YME1L KD Cat OV* (;*hmlΔ-Gal4>UAS-GFP/UAS-Cat;UAS-YME1L RNAi/+*) LGs (Figures 4G,H’) did not have primary lobe histolysis and were comparable to wild type (Figures 4A–C’,G,H’). Posterior lobes had significantly lower number of DAPI^+^ cells and no Hml^+^ cells. Further, PPO + crystal cell numbers were also rescued in *AAA* ^*+*^ *KD* LGs upon NAC treatment and catalase overexpression (Supplementary Figure S7A–K) Hence, pharmacological or genetic reduction of ROS levels in *AAA* ^*+*^ *KD* flies, indicate that ROS levels act as an important systemic effector downstream of AAA^+^ proteases in the regulation of *Drosophila* hematopoiesis.

### AAA^+^ proteases regulate hematopoiesis through yorkie activation

Among other signaling pathways, ROS levels were shown to activate *Drosophila* Yorkie or its mammalian ortholog YAP1 (Yes-associated protein 1) in *Drosophila* enterocytes and human glioma cell lines (Dixit et al., 2014; Nagai et al., 2021), a molecule which has been shown to be directly upregulating tissue proliferation (Milton et al., 2014; Snigdha et al., 2019). Since, ROS levels emerged to be critical in the *AAA* ^*+*^ *KD* LG phenotypes, we hypothesized that the ROS-Yorkie axis may be a key mechanism by which AAA^+^ proteases mediate LG homeostasis.

**FIGURE 5:**
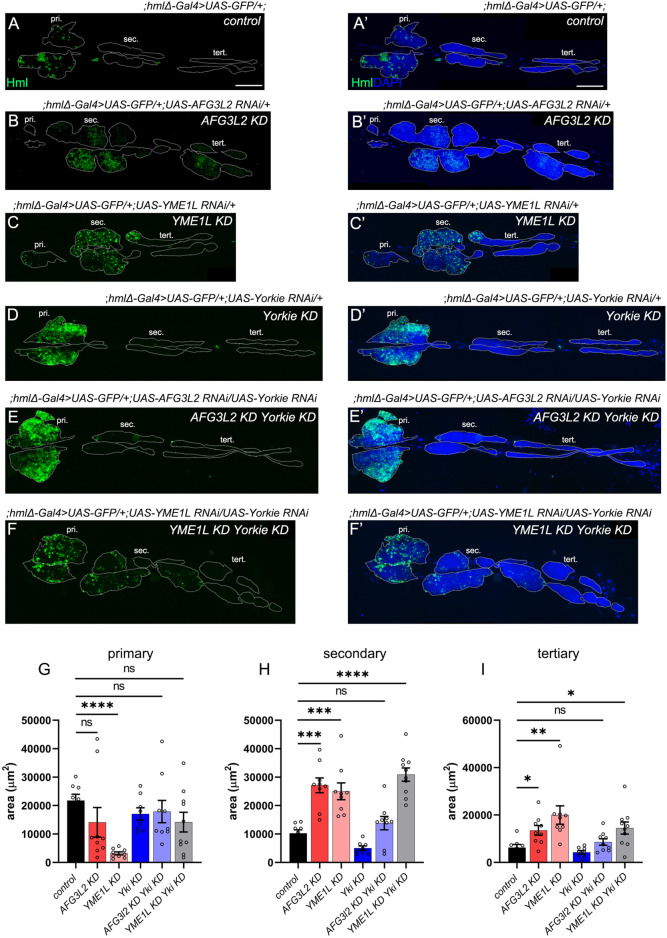
AAA^+^ proteases regulate hematopoiesis through Yorkie activation **(A–C’)** Lymph gland lobes of *control* (;*hmlΔ-Gal4>UAS-GFP/+;)* **(A,A’)**, *AFG3L2 KD (;hmlΔ-Gal4>UAS-GFP/+;UAS-AFG3L2 RNAi/+)* **(B,B’)** and *YME1L KD (;hmlΔ-Gal4>UAS-GFP/+;UAS-YME1L RNAi/+)* **(C,C’)** genotypes labelled with differentiation marker Hml (green) and nuclear marker DAPI (blue). **(D,D’)** Lymph gland lobes of *Yorkie KD (hmlΔ-Gal4>UAS-GFP/+;UAS-Yorkie RNAi/+)* samples, marked with Hml (green) and DAPI (blue). **(E-F’)** Lymph gland lobes of *AFG3L2 KD Yorkie KD* (;*hmlΔ-Gal4>UAS-GFP/+;UAS-AFG3L2 RNAi/UAS-Yorkie RNAi*) and *YME1L KD Yorkie KD* (;*hmlΔ-Gal4>UAS-GFP/+;UAS-YME1L RNAi/UAS-Yorkie RNAi*) samples, marked with Hml (green) and DAPI (blue). **(G–I)** Quantification of area (µm^2^) of the primary **(G)**, secondary **(H)** and tertiary **(I)** lobes of *control* (;*hmlΔ-Gal4>UAS-GFP/+;)* (N–8), *AFG3L2 KD (;hmlΔ-Gal4>UAS-GFP/+;UAS-AFG3L2 RNAi/+)* (N–9), *YME1L KD (;hmlΔ-Gal4>UAS-GFP/+;UAS-YME1L RNAi/+)* (N–9), *Yorkie KD (;hmlΔ-Gal4>UAS-GFP/+;UAS-Yorkie RNAi/+)* (N–8), *AFG3L2 KD Yorkie KD* (;*hmlΔ-Gal4>UAS-GFP/+;UAS-AFG3L2 RNAi/UAS-Yorkie RNAi*) (N-9) and *YME1L KD Yorkie KD* (;*hmlΔ-Gal4>UAS-GFP/+;UAS-YME1L RNAi/UAS-Yorkie RNAi*) (N-10) genotypes. Images represented are maximum intensity projections of individual slices. White dotted lines are used to mark the lymph gland lobes (pri, primary lobes; sec, secondary lobes; tert, tertiary lobes). Error bars represent SEM. Statistical significance was estimated using Students’ t-test with Welch’s correction (ns–nonsignificant, *p < 0.05, **p < 0.01, ***p < 0.001, ****p < 0.0001). Scale bars – 100 µm.

Hemocyte-specific depletion of Yorkie (Yorkie KD - *hmlΔ-Gal4>UAS-GFP/+;UAS-Yorkie RNAi/+*) had no effect on LG progenitor differentiation compared to control (Figures 5A,A’,D,D’). Depleting *Yorkie* in *AFG3L2 KD* hemocytes (*hmlΔ-Gal4>UAS-GFP/+;UAS-AFG3L2 RNAi/UAS-Yorkie RNAi)* restored both DAPI^+^ cell number and hemocyte differentiation to control levels as indicated in Figures 5A–F and area analyses of the LG lobes of mentioned samples (Figures 5G–I). However, *YME1L KD Yorkie KD* (*hmlΔ-Gal4>UAS-GFP/+;UAS-YME1L RNAi/UAS-Yorkie RNAi*) was comparable to *YME1L KD* in DAPI + cell numbers and Hml + hemocytes (Figure 5). Taken together, our data show that Hippo signaling mediated by Yorkie is involved in AAA^+^ protease mediated regulation of LG maintenance.

## Discussion

Mitochondrial homeostasis is critical for regulating diverse aspects of hematopoiesis, including quiescence of HSCs and other blood progenitors, proliferation, differentiation, and lineage bias (Snoeck, 2017; Filippi and Ghaffari, 2019; Papa et al., 2019). Mitochondrial properties including ROS levels, metabolism, calcium handling and morphology controlled by proteins such as Mfn2 (Mitofusin-2)/Marf, Drp1 (Dynamin-related protein 1), MTCH2 (Mitochondrial carrier homolog 2) and BID (BH3 interacting-domain death agonist) play decisive roles in hematopoiesis (Ray et al., 2021; Maryanovich et al., 2012; Maryanovich et al., 2015; Luchsinger et al., 2016). Here, our data demonstrate that ubiquitous mitochondrial proteome quality regulators AAA^+^ proteases AFG3L2 and YME1L are essential for *Drosophila* blood cell homeostasis. Although the expression levels of the mentioned proteases have been shown to be drastically low in the progenitors and prohemocyte pool compared to that of plasmatocytes according to the Fly hematopoiesis atlas (Cho et al., 2020), their depletion from progenitors caused severe larval lethality underscoring their absolute importance in the survival of the organisms beyond third instar stage. As we have used three different Gal4 drivers to target the progenitor pool of which the *tep4-Gal4* driver being extremely specific to core progenitors of the primary lobe flanking the dorsal vessel, the lethality seems to be specific and not arising from other tissues. While this may be expected, AAA^+^ proteases in the LG progenitors and PSC cells are critical for survival only beyond third instar stage (Supplementary Figure S2A–E). YME1L in LG progenitors and PSC cells appears to have a more pronounced impact on organismal survival than AFG3L2. Apart from forming homohexameric complexes, AFG3L2 can heterohexamerise with SPG7 proteins to form the m-AAA protease (Koppen et al., 2007). Since i-AAA protease complexes comprise of homohexamers of YME1L only, its depletion may not be compensated by other mechanisms. This may explain the severity of phenotypes following YME1L depletion. A recent study demonstrated that YME1L depletion in murine neural stem and progenitor cells leads to loss of quiescence and stem cell exhaustion due to mitochondrial proteome rewiring (Wani et al., 2022), underscoring its importance in progenitor maintenance. Further, since AFG3L2 depletion is tolerated in the core progenitors and PSC cells, deciphering its role in those cells would unravel canonical functions and mechanisms of mitochondrial AAA^+^ protease-mediated blood progenitor maintenance.

**FIGURE 6:**
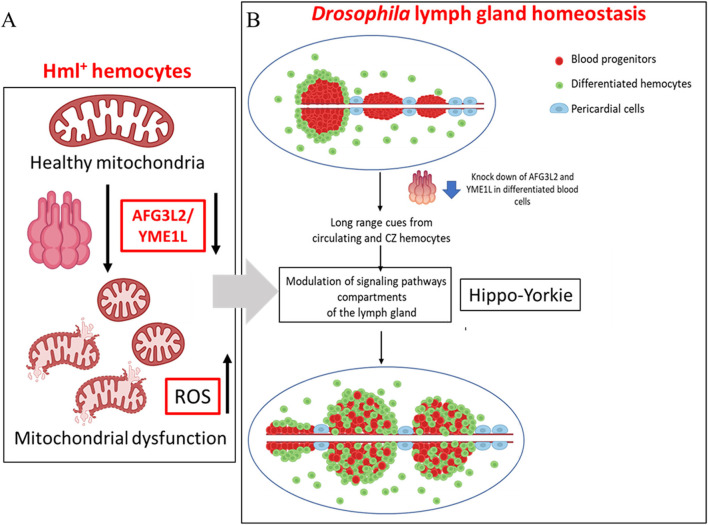
Mitochondrial AAA^+^ proteases AFG3L2 and YME1L regulate *Drosophila* hematopoiesis through their functions in differentiated hemocytes **(A)** Hemocyte specific depletion of AFG3L2 and YME1L leads to mitochondrial dysfunction as indicated by fragmented mitochondria and high cellular ROS levels. **(B)** Depletion of AFG3L2 and YME1L in hemocytes lead to precocious differentiation of lymph gland progenitors and hyperplasia of the posterior lobes, possibly through systemic cues emerging from the hemocytes. Signaling pathways including Hippo-Yorkie act downstream of AFG3L2 to regulate hematopoiesis.

Here, our results show that hemocyte-specific depletion of AFG3L2 and YME1L leads to gross defects in LG homeostasis as depicted schematically in Figure 6. In *AAA* ^*+*^ *KD* larvae, LG progenitors differentiate precociously from second instar stage (72 h AEL) onwards, while the differentiated hemocytes start dissociating from the LG primary lobes by mid-third instar (96 h AEL). Through our developmental mapping of the *AAA* ^*+*^ *KD* LGs, we observed premature differentiation of primary lobe progenitors in earlier stages, followed by differentiation of the posterior lobes at later stages, in agreement with our recent studies demonstrating progenitors to be developmentally heterogeneous across LG lobes (Rodrigues et al., 2021a). The posterior lobes undergo hyperplasia and extensive differentiation, but the cells do not dissociate from the lobes (Supplementary Figures S4A–D, Figure 1G–K). The evident heterogeneity in phenotypes among the progenitors across different lobes can be explained by the developmental immaturity of the posterior lobe progenitors, as we have demonstrated previously (Rodrigues et al., 2021a; Ray et al., 2021). ECM (extracellular matrix) proteins including Perlecan, Collagen-IV and their cell-surface receptors, the integrins regulate *Drosophila* hematopoiesis through their roles in maintaining tissue architecture, cell-cell adhesion, ligand availability, and signaling pathway modulations (Kim and Choe, 2014; Grigorian et al., 2011; Khadilkar et al., 2020), although the components are differentially expressed across LG lobes (Rodrigues et al., 2021a). Hence, one possibility is that the heterogenous phenotypes of lobe integrity in *AAA* ^*+*^ *KD* LGs could be through disrupted ECM maintenance, which warrants further investigation.

In *AAA* ^*+*^ *KD* larvae, there were extensive differentiation to Hml + hemocytes with a concomitant decrease in the DomeMesoBFP + progenitor proportion in the secondary lobes indicating premature differentiation of the posterior progenitors which are otherwise resilient to differentiation cues. Further, PSC cell numbers were also found to be mildly increased which highlights the differential impact of AAA protease depletion on lymph gland cell types. The PSC may not be proliferative, as suggested by H3P staining data. Whether the cells are defective or have any other role remains to be ascertained. We observed precocious differentiation of the progenitors to all three hemocyte lineages, including lamellocytes which only emerge during wasp infestation and genetic modulations of certain signaling pathways. This suggested the involvement of AAA^+^ proteases in systemic regulation of progenitor maintenance, even when depleted only in the differentiated Hml^+^ hemocytes. Genetic upregulation of ROS levels in the progenitors by depleting the, ETC., complex I protein ND75 elicits similar phenotypes of unbiased differentiation in *Drosophila* LGs (Owusu-Ansah and Banerjee, 2009). Here, our data demonstrate that reducing ROS levels systemically or in a hemocyte-specific manner, can reverse the *AAA* ^*+*^ *KD* LG phenotypes (Figure 6). This indicates that high ROS levels in differentiated hemocytes can also regulate progenitor maintenance and consequently LG homeostasis.

Although, the mitochondrial phenotypes of AFG3L2 and YME1L depletion in different contexts appear similar owing to their non-specific roles in regulating the mitochondrial proteome, intricate mechanisms employed by these proteases in impinging upon mitochondrial homeostasis exist. For example, AFG3L2 has been shown to regulate mitochondrial calcium levels by governing the turnover of EMRE, the gatekeeper of mitochondrial calcium uniporter (MCU). In the absence of AFG3L2, mitochondria undergo calcium overload leading to apoptosis and neuronal loss in mice (König et al., 2016). Depletion of YME1L in murine NSPCs results in loss of the progenitor pool and premature differentiation, which could be partially rescued by depleting OMA1, resulting in restoration of s-OPA1/l-OPA1 balance, suggesting the role of mitochondrial morphology under the control of YME1L to be one of the critical mechanisms in this process (Wani et al., 2022). Therefore, since the AAA^+^ proteases regulate a host of other mitochondrial properties including calcium handling, morphology and bioenergetics, individual effects of these properties under the control AAA^+^ proteases in *Drosophila* hematopoiesis merit detailed investigation.

Multiple signaling pathways including JNK, JAK/STAT and Hippo-Yorkie regulate *Drosophila* blood progenitor maintenance and differentiation (Milton et al., 2014; Owusu-Ansah and Banerjee, 2009; Anderson et al., 2017). The *AAA* ^*+*^ *KD* LG phenotypes of differentiation, were similar to that of the LG phenotypes observed upon inactivating the Hippo pathway (Milton et al., 2014), which is reported to regulate organ size and tissue homeostasis (Yu et al., 2015). Ablating Warts, one of the kinases involved in the pathway, led to precocious differentiation of blood progenitors, which could be rescued by simultaneously depleting the downstream transcriptional co-activator, Yorkie (Milton et al., 2014). Further, ROS levels were shown to activate *Drosophila* Yorkie or its mammalian ortholog YAP1 (Yes-associated protein 1) in *Drosophila* enterocytes and human glioma cell lines (Dixit et al., 2014; Nagai et al., 2021). Taken together, these studies suggested that the AAA^+^ proteases may impinge their effects on LG homeostasis through ROS-mediated control of Hippo. Here, our data show that *AFG3L2 KD* phenotypes could be reversed by ablating Yorkie in the hemocytes, but not that of *YME1L KD*, suggesting potential compensatory mechanisms of AFG3L2 depletion. Further, since ROS levels have ubiquitous effects on multiple factors regulating hematopoiesis, the phenotypes of *YME1L KD* LGs could be through other mechanisms under the control of ROS.

In this study, we provide evidence that the proteases in hemocytes impact blood progenitor maintenance and differentiation via controlling ROS levels. Furthermore, our data demonstrate the involvement of Hippo-Yorkie pathway as a critical effector downstream of the AAA^+^ proteases, in regulating hematopoiesis. Mitochondrial AAA^+^ proteases have been extensively characterized in regulating mitochondrial properties during various neuronal disorders. Studies on *Drosophila* ROS and YME1L have also underscored their roles in ageing and neurodegeneration associated with mitochondrial aberrations. However, potential roles of these proteases in non-cell autonomous regulation of progenitor fate through signaling pathways were unknown. This study provides novel insights into previously unexplored roles and mechanisms of mitochondrial AAA^+^ proteases in maintaining blood cell homeostasis through ROS-mediated control of signaling pathways.

## Methods and materials

### *Drosophila* stocks and genetics

*Dome-Gal4,UAS-2xEGFP, Antp-Gal4* were gifts from Utpal Banerjee. *tep4-Gal4,UAS-GFP* was gifted by Lucas Waltzer. *pCol85-Gal4,UASmCD8-GFP* was gifted by Michele Crozatier, *HmlΔGal4,UAS-2xEGFP*, *UAS-catalase (w* (Mochizuki-Kashio et al., 2021)*; P{w[+mC] = UAS-Cat.A}2) (BDSC #24621), domeMeso-BFP and domeMeso-Gal4,UAS-GFP* were gifts from Tina Mukherjee. *UAS-Yorkie-RNAi (y* (Mochizuki-Kashio et al., 2021) *v* (Mochizuki-Kashio et al., 2021)*; P{y[+t7.7] v[+t1.8] = TRiP.HMS00041}attP2) (BDSC #34067)* was gifted by Carmen Coelho. *UAS-AFG3L2-RNAi (y* (Mochizuki-Kashio et al., 2021) sc[*] v (Mochizuki-Kashio et al., 2021) *sev* (König et al., 2016)*; P{y[+t7.7] v[+t1.8] = TRiP.HMS01331}attP2)* (BDSC #34343) and *UAS-YME1L-RNAi (y* (Mochizuki-Kashio et al., 2021) *v* (Mochizuki-Kashio et al., 2021)*; P{y[+t7.7] v[+t1.8] = TRiP.HMC03303}attP2)* (BDSC #51752) were obtained from Bloomington *Drosophila* Stock Center (BDSC). *Canton-S* was used as the wild type strain. The stocks were maintained under standard rearing conditions at 2 °C, unless specified otherwise.

RNAi knockdown was validated by qRT-PCR and mitochondrial morphology phenotype.

For NAC treatment, fly food was mixed with NAC (A9165, Sigma Aldrich) at a concentration of 10 mg/mL and parent crosses were cultured for examining emerging larvae at wandering third instar larval stage.

### Neutral red staining and blinded quantification of LG morphology

Lymph glands were dissected in PBS (phosphate buffered saline) and fixed using 4% PFA (– 158,127, Sigma Aldrich, India) (paraformaldehyde), followed by washing with PBS. The LGs were incubated in 0.2% neutral red solution (72,210-25G, Merck, Germany) in PBS for 5 min, after washing with PBS. After washing post-incubation, they were imaged in Olympus SZX12 stereozoom microscope (OLY-SZX12-B, Olympus, Japan) at 4X magnification for blinded quantification of LG morphology by two independent researchers to qualitatively classify the LG lobes of randomly numbered samples into three categories–*regular* (as observed under normal conditions), *disintegrated* (histolyzing and histolyzed lobes) and *tumorous* (abnormal lobes with tumorous growths) Representative images were acquired in Leica DMi8 (Germany) microscope.

### Immunofluorescence staining and microscopy

For LG analyses, wandering third instar larvae (144 h AEL) were dissected in PBS, fixed in 4% PFA for 20 min, followed by washing in PBS. The LGs were then permeabilized using 0.3% Triton X-100 (X100-5ML, Merck, Germany) for 20 min and washed in PBS. After permeabilization, the LGs preps were incubated in 20% normal goat serum (RM10701,HiMedia, India) for 1 h. Primary antibody dilutions were then added to the samples and incubated overnight at 4 °C. Primary antibody incubation was followed by washes in PBS and incubation with appropriate secondary antibodies for 2 hours. Secondary antibody incubation was followed by extensive washes in PBS. The samples were then mounted in 70% glycerol (Q24505, ThermoFisher Scientific, India) containing 4′6-diamidino-2-phenylindole (DAPI) (D1306, ThermoFisher Scientific, United States) and imaged in Zeiss LSM880 confocal microscope at 20X magnification. To visualize the entire lymph gland, multiple images were captured from the anterior-posterior axis and stitched manually.

For circulating hemocyte immunostaining, individual larvae of each genotype were bled in 50 µL of PBS and allowed to adhere to coverslip bottomed 96 well plates (P96-15H-N, CellVis, United States) for 45 min, following which, the cells were fixed using 4% PFA for 15 min. Fixation was followed by washing and permeabilization with 0.4% NP40 (Nonidet P-40; 492016, Sigma Aldrich, India). The cells were then washed again and incubated with 2 mg/mL BSA (bovine serum albumin; MB083, Himedia, India) for 1 h. Blocking was followed by primary antibody incubation at 4 °C overnight. Primary antibody incubation was followed by washes in PBS and incubation with appropriate secondary antibodies for 2 hours. The cells were then incubated in a DAPI:PBS solution for 10 min, and washed. Imaging was performed in Zeiss LSM880 confocal microscope at 40X magnification. All antibody dilutions were made in PBS.

### Antibodies

Mouse anti-Antennapedia (1:20, 4C3, DSHB), anti-P1 antibody (1:20) and anti-L1 abtibody (kind gifts from Istvan Ando) mouse anti-ProPO antibody (1:20, self-generated through Bioneeds India Private Limited), and rabbit anti-H3P antibody (1:100, Abcam ab5176, Boston, United States). Phalloidin was conjugated to Alexa-633 (A-22284, Thermofisher Scientific, United States) and secondary antibodies were Alexa-488 (A-11008 and A-11001, Thermofisher Scientific, United States), Alexa-568 (A-11011 and A-11004, Thermofisher Scientific, United States), or Alexa-633 (A-21050 and A-21070, Thermofisher Scientific, United States) conjugated.

### Lethality, pupariation, eclosion and life span assays

For lethality assays, *UAS-AFG3L2-RNAi and UAS-YME1L-RNAi* were crossed with progenitor and PSC-specific drivers. GFP^+^ or non-GFP^+^ wandering third instar larvae were counted and the proportions with total larvae collected were quantified for all drivers except *antp-Gal4*. For lethality using *antp-Gal4* driver, the Gal4 line was crossed with *Canton-S* flies which served as the control, and non-tubby flies were counted in control and AAA^+^ depleted genotypes. The experiments were repeated at least thrice, with at least 10 larvae in each replicate.

For pupariation rate, fly crosses were kept for 6 h for egg laying in glass. After 120 h of AEL, 3rd instar larvae were counted and monitored for pupariation. The experiment was repeated thrice with at least 40 flies in each replicate. Once pupariated, the flies were monitored for eclosion and the rates were compared.

For life span assays, male flies post 3-day eclosion were collected and maintained in sterile fly food bottle at 25 °C in cornmeal agar medium. Flies were transferred in fresh bottles every 3 days to avoid any pathogenic challenge. Five sets, each with minimum 12 flies were monitored daily, and natural deaths were recorded. Median survival rate was compared.

### Dihydroethidium (DHE) staining for cellular ROS levels

For visualizing and quantifying cellular ROS levels, larvae were bled in 50 µL Schneider’s media (21720-024, ThermoFisher Scientific, Paisley, Scotland, United Kingdom) and allowed to adhere onto 96-well coverslip bottomed dishes for 45 min. The cells were then incubated with 5 µM DHE (D11347, ThermoFisher Scientific, United States) in Schneider’s media for 15 min. Incubation was followed by washing with Schneider’s media. The cells were imaged live in a Zeiss LSM880 confocal microscope attached to a sample heater (25 °C).

### Live imaging of mitochondria and morphology analysis

For live imaging of mitochondria, 4-5 larvae were bled in 50 µL Schneider’s media, and allowed to adhere onto 96-well coverslip-bottomed dishes for 45 min. After washing the cells once with 1X PBS, MitoTracker Deep Red (M22426, ThermoFisher Scientific, United States) at a concentration of 5 nM made in Schneider’s media was added and incubated for 15 min at 25 °C. After washing with Schneider’s media twice, the cells were imaged live in a Zeiss LSM880 confocal microscope attached to a sample heater (25 °C) to control temperature. Images of different fields were acquired, and cells were analysed individually. Mitochondrial morphology analysis was performed on FIJI (https://imagej.net/software/fiji/downloads) with the MiNA (Mitochondrial Network Analysis) plugin (https://github.com/StuartLab/MiNA) as described elsewhere [50].

### Image processing and analysis

Maximum intensity projections were made from complete Z-stacks and manually stitched for representative figures. Images were processed uniformly for brightness and contrast using Adobe Photoshop CS5 (Adobe Inc., United States). White dotted lines indicate lymph gland lobe boundaries. Mean fluorescence intensities (MFI) were measured using ImageJ software. Cell count analyses using various markers were performed using the spots module in IMARIS (Oxford Instruments, United Kingdom). Quantifications were performed for the primary, secondary, and tertiary lobes individually.

### Statistical analysis

For all the experiments, statistical significance was deduced by Students’ t-test with Welch’s correction except for life span assay. For life span assay, Mantel-Cox log-rank test was used. Graphs and statistical analyses were performed in GraphPad Prism 8.0 (Dotmatics, United States). Statistically significant differences were indicated by *p < 0.05, **p < 0.01, and ***p < 0.001 and ns indicates nonsignificant. Error bars represent standard error of mean (SEM).

## Data Availability

The original contributions presented in the study are included in the article/Supplementary Material, further inquiries can be directed to the corresponding author.
