# Supplementary figures and images for: Mitochondrial AAA+ protease activity uncovers differential sensitivity of Drosophila blood cell lineages to systemic cues
Source: Front Cell Dev Biol. 2025 Nov 24;13:1606805. doi: 10.3389/fcell.2025.1606805 (PMC12682893; doi:10.3389/fcell.2025.1606805)

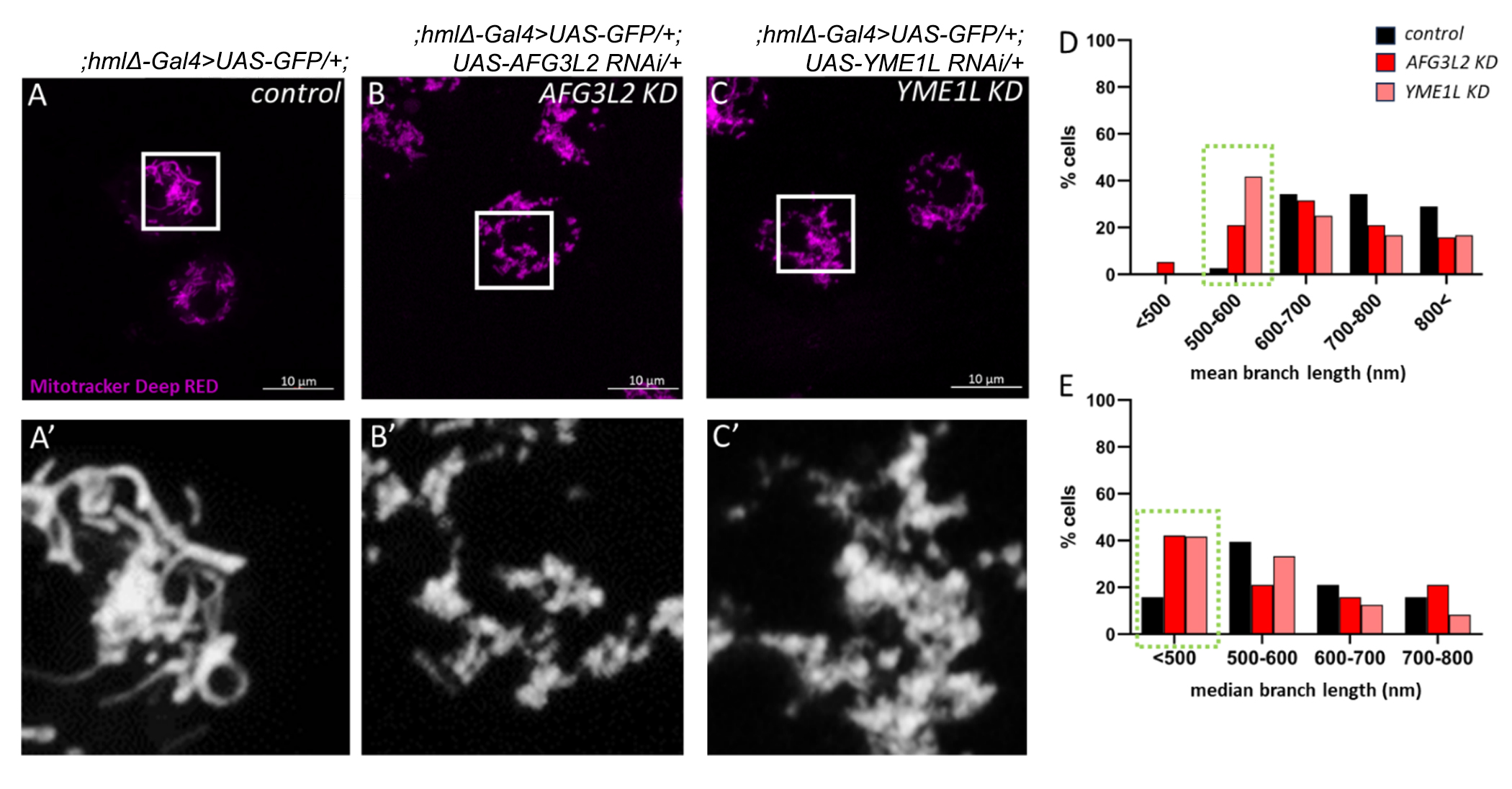

Supplement: Supplementary file 1 [file Image3.jpeg]

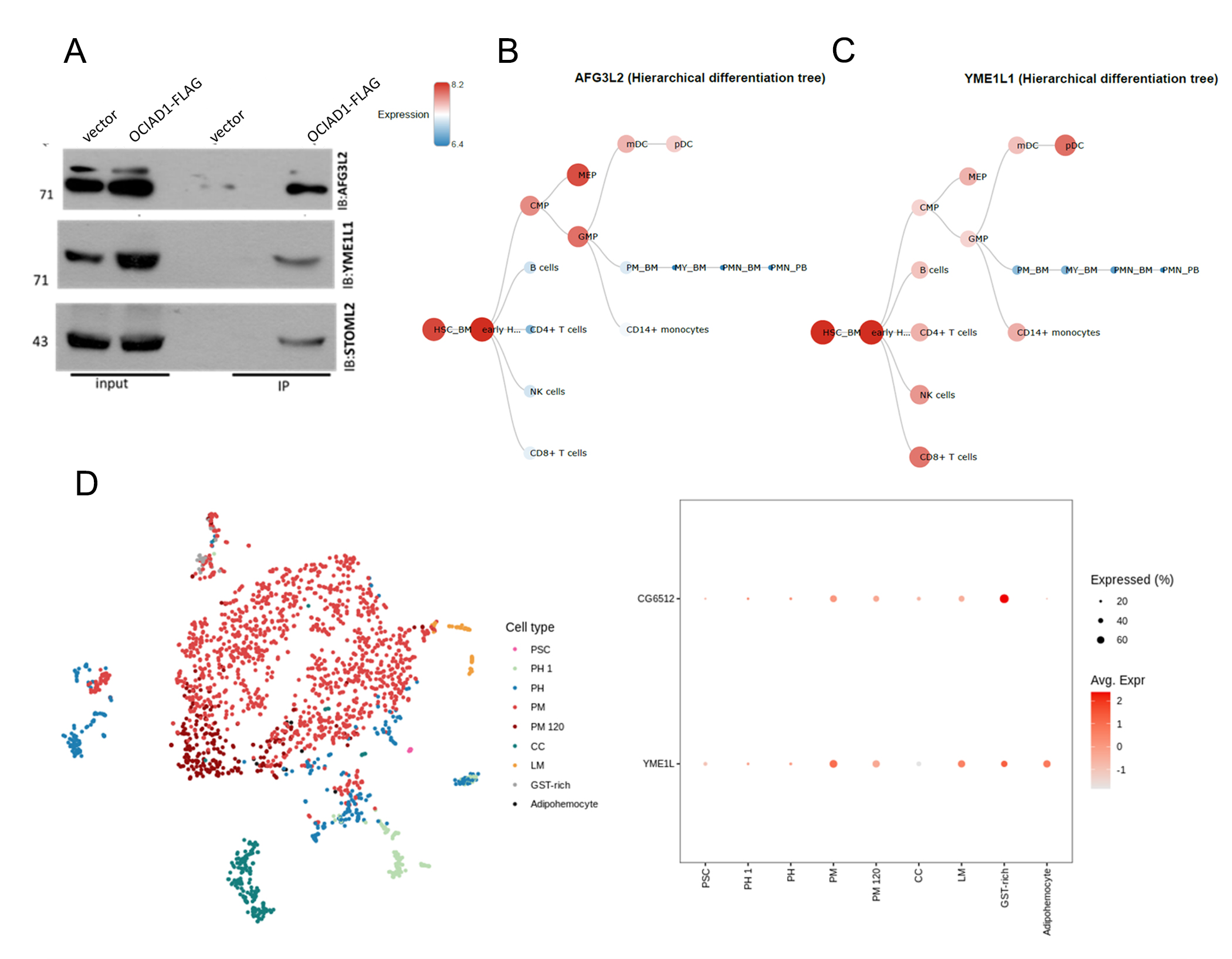

Supplement: Supplementary file 2 [file Image1.jpeg]

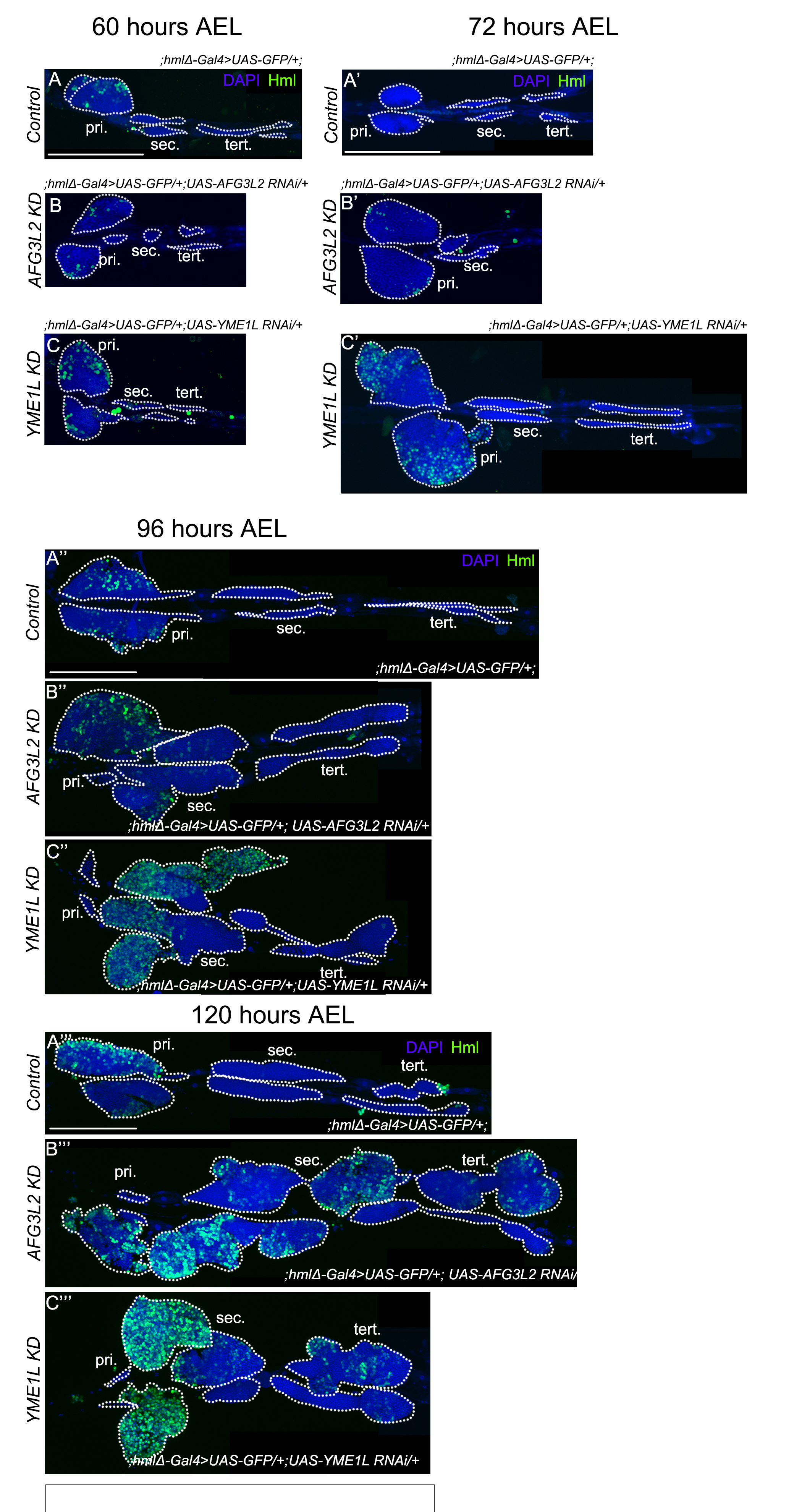

Supplement: Supplementary file 3 [file Image4.jpeg]

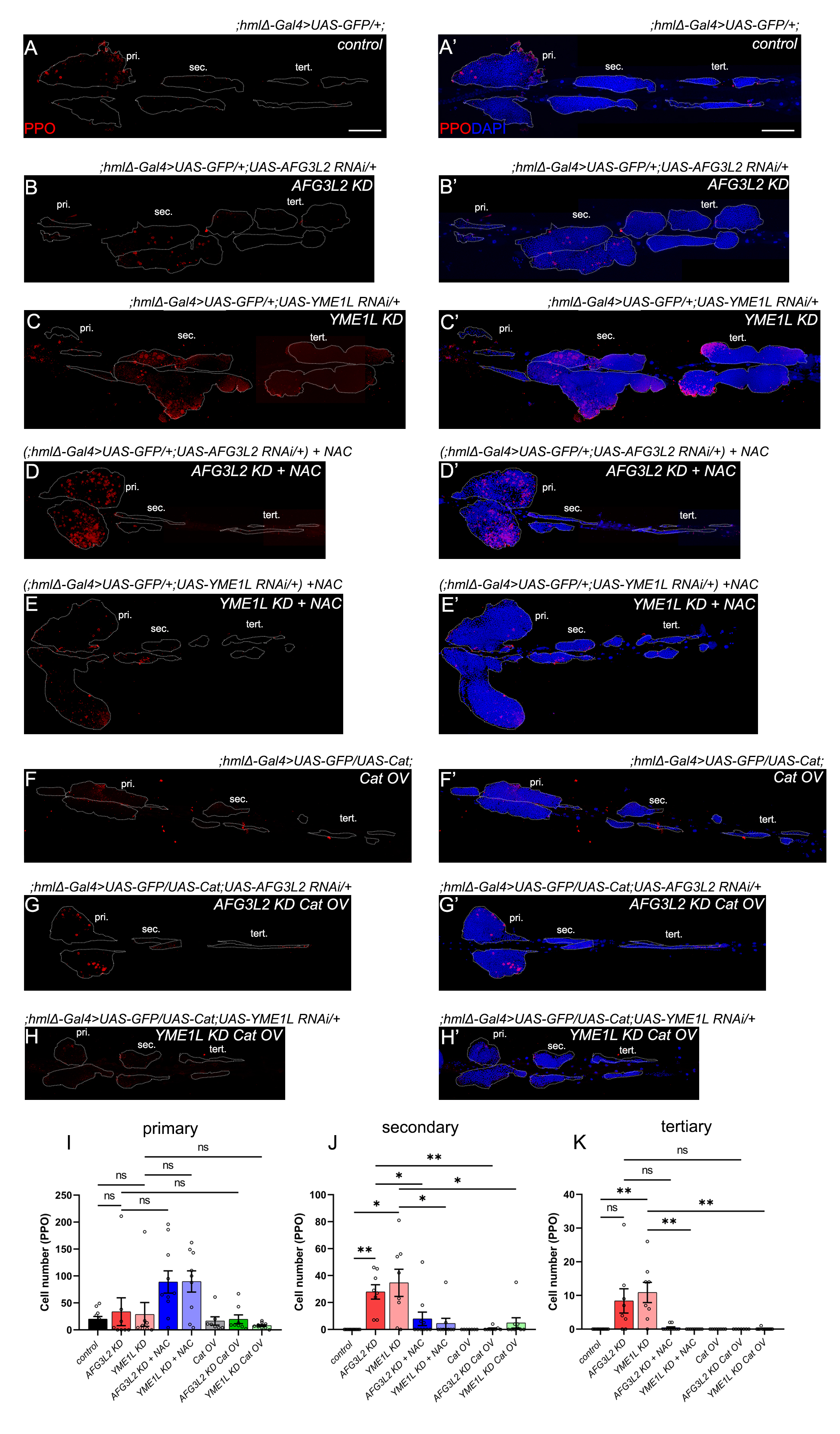

Supplement: Supplementary file 4 [file Image7.jpeg]

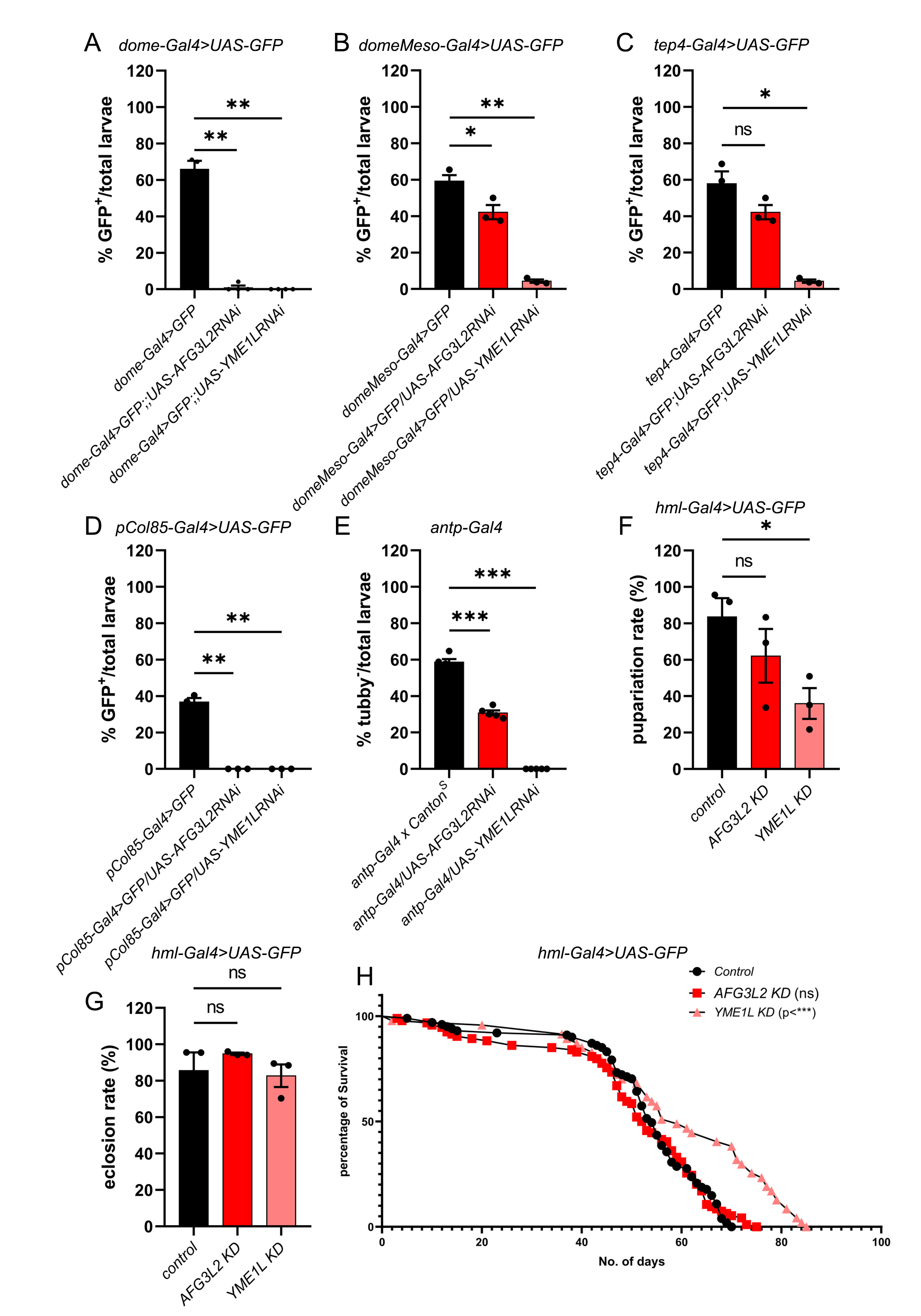

Supplement: Supplementary file 5 [file Image2.jpeg]

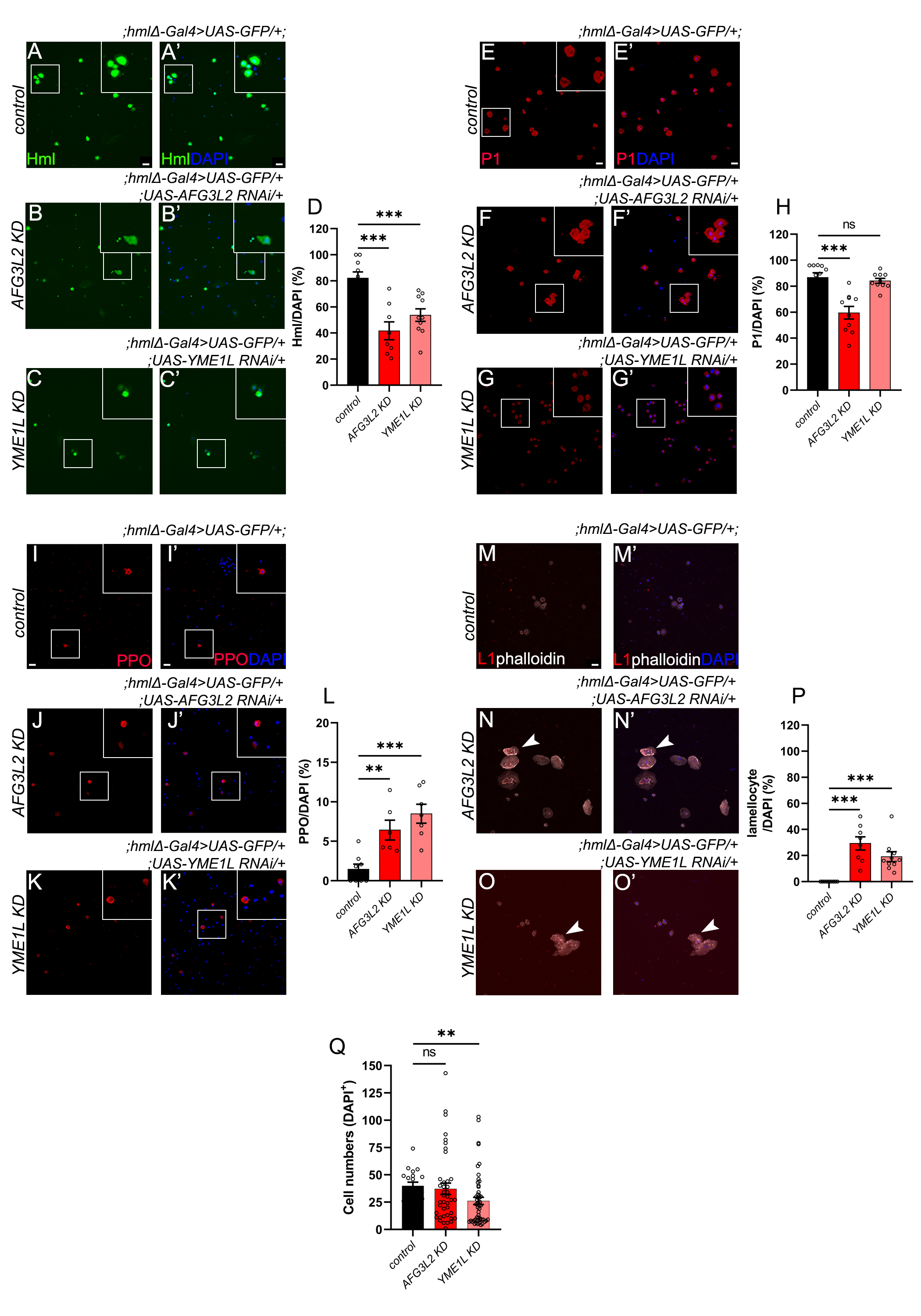

Supplement: Supplementary file 6 [file Image5.jpeg]

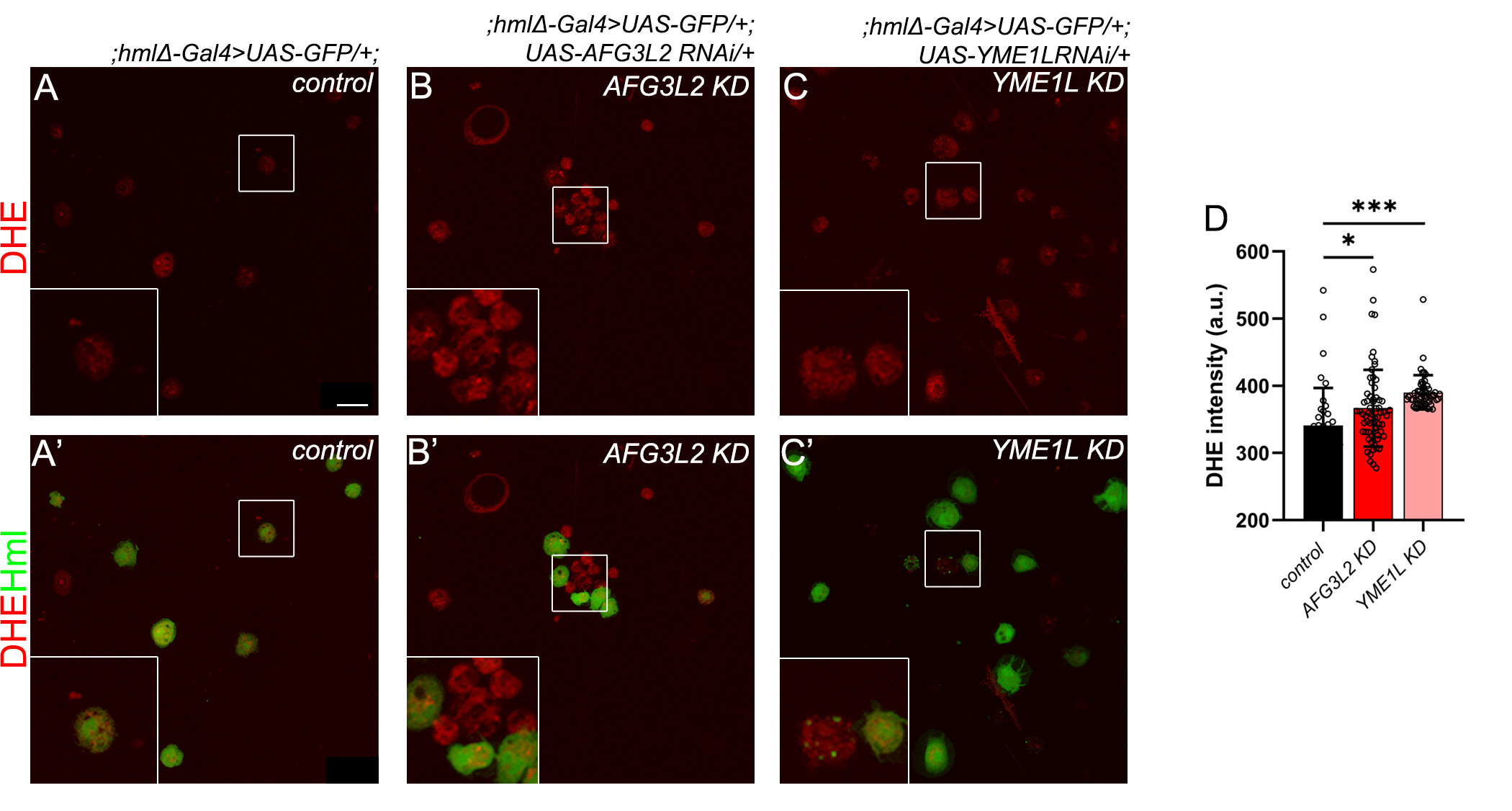

Supplement: Supplementary file 8 [file Image6.jpeg]
