# Supplementary material for: Mitochondrial AAA+ protease activity uncovers differential sensitivity of Drosophila blood cell lineages to systemic cues
Source: Front Cell Dev Biol. 2025 Nov 24;13:1606805. doi: 10.3389/fcell.2025.1606805 (PMC12682893; doi:10.3389/fcell.2025.1606805)
Supplement: Supplementary file 7 [file DataSheet1.docx]

**Mitochondrial AAA^+^ protease activity uncovers differential sensitivity of *Drosophila* blood cell lineages to systemic cues.**

Rajarshi Batabyal^1^, Aman Sharma^1^ and Maneesha S Inamdar^1,2*^

^1^Jawaharlal Nehru Centre for Advanced Scientific Research, Bangalore, India; ^2^Institute for Stem Cell Science and Regenerative Medicine (BRIC-InStem), Bangalore, India.

**Supplementary Figure Legends:**

**Fig S1. AFG3L2 and YME1L interact with OCIAD1 and are differentially expressed in blood cells in humans.**

(A) Interaction between OCIAD1-FLAG and AFG3L2, YME1L and the scaffold protein STOML2

(B-C) Expression pattern of AFG3L2 and YME1L normal hematopoiesis, as shown in databases [24]. (D) Expression pattern of AFG3L2 (CG6512) and YME1L in Drosophila hematopoiesis as observed in the Fly Hemocyte Atlas [27]

**Fig S2**. **AAA^+^ proteases regulate organismal health through their functions in blood cells.**

(A-E) Quantification of lethality by third instar larval stages upon AFG3L2 and YME1L depletion in progenitors using *dome-Gal4>UAS-GFP* (A), *domeMeso-Gal4>UAS-GFP* (B) and *tep4-Gal4>UAS-GFP* (C) or in PSCs using *pCol85Gal4>UAS-GFP* (D) and *antpGal4* (E). (N≥3, n≥8 3^rd^ instar wandering larvae)

(F) Quantification of pupariation rates and (G) eclosion rates of *control* (;*hmlΔ-Gal4>UAS-GFP/+;)*, *AFG3L2 KD (;hmlΔ-Gal4>UAS-GFP/+;UAS-AFG3L2 RNAi/+)* and *YME1L KD (;hmlΔ-Gal4>UAS-GFP/+;UAS-YME1L RNAi/+) flies* (N=3, n≥40)

(H) Survival curve of *control* (;*hmlΔ-Gal4>UAS-GFP/+;)*, *AFG3L2 KD (;hmlΔ-Gal4>UAS-GFP/+;UAS-AFG3L2 RNAi/+)* and *YME1L KD (;hmlΔ-Gal4>UAS-GFP/+;UAS-YME1L RNAi/+)* flies (N=5, n≥12 adult flies)

Error bars represent SEM. Statistical significance was estimated using Students’ t test with Welch’s correction in all the cases except in life span assay (H) where Log Rank (Mantel-Cox) test was used(ns – nonsignificant, *p<0.05, **p<0.01, ***p<0.001).

**Fig S3. AFG3L2 and YME1L KD larvae have elevated percentage of circulating hemocytes with fragmented mitochondria**.

(A-C’) Mitotracker Deep Red stained hemocytes in circulation of *control* (;*hmlΔ-Gal4>UAS-GFP/+;)* (A,A’), *AFG3L2 KD (;hmlΔ-Gal4>UAS-GFP/+;UAS-AFG3L2 RNAi/+)*  (B,B’) and *YME1L KD (;hmlΔ-Gal4>UAS-GFP/+;UAS-YME1L RNAi/+)* (C,C’) larvae (scale bar – 10 µm).

(D and E) Quantification of percentages of cells with mean branch lengths (nm) (D) and median branch lengths (nm) (E) binned into mentioned ranges of control (38 cells), AFG3L2 KD (19 cells) and YME1L KD (24 cells) hemocytes.

**Fig S4**. **AAA^+^ proteases depletion phenotypes in hemocytes appear progressively through development.**

(A-D) Hml^+^ (green) hemocytes and DAPI^+^ (blue) cells in *control* (;*hmlΔ-Gal4>UAS-GFP/+;)*, *AFG3L2 KD (;hmlΔ-Gal4>UAS-GFP/+;UAS-AFG3L2 RNAi/+)* and *YME1L KD (;hmlΔ-Gal4>UAS-GFP/+;UAS-YME1L RNAi/+)*lymph glands at 60 hours (after egg laying) AEL (A), 72 hours AEL (B), 96 hours AEL (C) and 120 hours AEL (D).

Images represented are maximum intensity projections of individual slices. White dotted lines are used to mark the lymph gland lobes (pri.- primary lobes, sec.- secondary lobes, tert.-tertiary lobes). Scale bars – 200 µm.

**Fig S5**. **AAA^+^ proteases depletion in hemocytes lead to aberrant composition of circulating hemocytes.**

(A-C’) Hml^+^ (green) hemocytes and DAPI^+^ (blue) cells in circulation of *control* (;*hmlΔ-Gal4>UAS-GFP/+;)*, *AFG3L2 KD (;hmlΔ-Gal4>UAS-GFP/+;UAS-AFG3L2 RNAi/+)* and *YME1L KD (;hmlΔ-Gal4>UAS-GFP/+;UAS-YME1L RNAi/+)* larvae.

(D) Quantification of Hml^+^ fraction in circulating hemocytes of *control* (;*hmlΔ-Gal4>UAS-GFP/+;)*, *AFG3L2 KD (;hmlΔ-Gal4>UAS-GFP/+;UAS-AFG3L2 RNAi/+)* and *YME1L KD (;hmlΔ-Gal4>UAS-GFP/+;UAS-YME1L RNAi/+)*.

(E-G’) P1^+^ (red) plasmatocytes and DAPI^+^ (blue) cells in circulation of *control* (;*hmlΔ-Gal4>UAS-GFP/+;)*, *AFG3L2 KD (;hmlΔ-Gal4>UAS-GFP/+;UAS-AFG3L2 RNAi/+)* and *YME1L KD (;hmlΔ-Gal4>UAS-GFP/+;UAS-YME1L RNAi/+)*.

(H) Quantification of P1^+^ fraction in circulating hemocytes of *control* (;*hmlΔ-Gal4>UAS-GFP/+;)*, *AFG3L2 KD (;hmlΔ-Gal4>UAS-GFP/+;UAS-AFG3L2 RNAi/+)* and *YME1L KD (;hmlΔ-Gal4>UAS-GFP/+;UAS-YME1L RNAi/+)*.

(I-K’) PPO^+^ (red) plasmatocytes and DAPI^+^ (blue) cells in circulation of *control* (;*hmlΔ-Gal4>UAS-GFP/+;)*, *AFG3L2 KD (;hmlΔ-Gal4>UAS-GFP/+;UAS-AFG3L2 RNAi/+)* and *YME1L KD (;hmlΔ-Gal4>UAS-GFP/+;UAS-YME1L RNAi/+)* larvae.

(L) Quantification of PPO^+^ fraction in circulating hemocytes of *control* (;*hmlΔ-Gal4>UAS-GFP/+;)*, *AFG3L2 KD (;hmlΔ-Gal4>UAS-GFP/+;UAS-AFG3L2 RNAi/+)* and *YME1L KD (;hmlΔ-Gal4>UAS-GFP/+;UAS-YME1L RNAi/+)* larvae.

(M-O’) L1^+^(red)phalloidin^high^(white) lamellocytes and DAPI^+^ (blue) cells in circulation of *control* (;*hmlΔ-Gal4>UAS-GFP/+;)*, *AFG3L2 KD (;hmlΔ-Gal4>UAS-GFP/+;UAS-AFG3L2 RNAi/+)* and *YME1L KD (;hmlΔ-Gal4>UAS-GFP/+;UAS-YME1L RNAi/+)* larvae.

(P) Quantification of L1^+^phalloidin^high^lamellocytes fraction in circulating hemocytes of *control* (;*hmlΔ-Gal4>UAS-GFP/+;)*, *AFG3L2 KD (;hmlΔ-Gal4>UAS-GFP/+;UAS-AFG3L2 RNAi/+)* and *YME1L KD (;hmlΔ-Gal4>UAS-GFP/+;UAS-YME1L RNAi/+)* larvae.

(Q) Quantification of DAPI^+^ hemocytes in circulation of *control* (;*hmlΔ-Gal4>UAS-GFP/+;)*, *AFG3L2 KD (;hmlΔ-Gal4>UAS-GFP/+;UAS-AFG3L2 RNAi/+)* and *YME1L KD (;hmlΔ-Gal4>UAS-GFP/+;UAS-YME1L RNAi/+)* larvae.

Error bars represent SEM. Statistical significance was estimated using Students’ t test with Welch’s correction (N≥9 larvae, ns – nonsignificant, *p<0.05, **p<0.01, ***p<0.001). Scale bars – 20 µm.

**Fig S6**. **AAA^+^ proteases depletion in hemocytes lead to increased cellular ROS levels in circulating hemocytes.**

(A-C’) Hml^+^ (green) hemocytes with ROS levels labelled by DHE (red) cells in circulation of *control* (;*hmlΔ-Gal4>UAS-GFP/+;)*, *AFG3L2 KD (;hmlΔ-Gal4>UAS-GFP/+;UAS-AFG3L2 RNAi/+)* and *YME1L KD (;hmlΔ-Gal4>UAS-GFP/+;UAS-YME1L RNAi/+)* larvae.

(D) Quantification of ROS levels as indicated by intensities of DHE fluorescence (depicted as arbitrary units) in circulating hemocytes of *control* (;*hmlΔ-Gal4>UAS-GFP/+;)* (n=40 cells), *AFG3L2 KD (;hmlΔ-Gal4>UAS-GFP/+;UAS-AFG3L2 RNAi/+)* (n=65 cells) and *YME1L KD (;hmlΔ-Gal4>UAS-GFP/+;UAS-YME1L RNAi/+)* (n=52 cells) larvae.

Error bars represent SEM. Statistical significance was estimated using Students’ t test with Welch’s correction ( ns – nonsignificant, *p<0.05, **p<0.01, ***p<0.001). Scale bars – 20 µm.

**Fig S7. Crystal cell numbers are rescued in AAA+ KD LGs upon NAC treatment and catalase overexpression**

(A-C’) Lymph gland lobes of *control* (;*hmlΔ-Gal4>UAS-GFP/+;)* (A-A’), *AFG3L2 KD (;hmlΔ-Gal4>UAS-GFP/+;UAS-AFG3L2 RNAi/+)* (B-B’) and *YME1L KD (;hmlΔ-Gal4>UAS-GFP/+;UAS-YME1L RNAi/+)* (C-C’) genotypes labelled with differentiation marker PPO (red) and nuclear marker DAPI (blue), without any treatment.

(D-E’) Lymph gland lobes of *AFG3L2 KD (;hmlΔ-Gal4>UAS-GFP/+;UAS-AFG3L2 RNAi/+) and YME1L KD* *(;hmlΔ-Gal4>UAS-GFP/+;UAS-YME1L RNAi/+)* samples, upon NAC treatment, marked with PPO (red) and DAPI (blue).

(F-F’) Lymph gland lobes of *Cat OV (;hmlΔ-Gal4>UAS-GFP/UAS-Cat;) samples*, marked with PPO (red) and DAPI (blue).

(G-H’) Lymph gland lobes of *AFG3L2 KD Cat OV (;hmlΔ-Gal4>UAS-GFP/UAS-Cat;UAS-AFG3L2 RNAi/+) and YME1L KD Cat OV (;hmlΔ-Gal4>UAS-GFP/UAS-Cat;UAS-YME1L RNAi/+)* samples, marked with PPO (red) and DAPI (blue).

(I - K) Quantification of PPO/DAPI^+^ cell numbers across the primary (I), secondary (J) and tertiary (K) lobes of *control* (;*hmlΔ-Gal4>UAS-GFP/+;)* (N – 11), *AFG3L2 KD* *(;hmlΔ-Gal4>UAS-GFP/+;UAS-AFG3L2 RNAi/+)* (N – 8), *YME1L KD* *(;hmlΔ-Gal4>UAS-GFP/+;UAS-YME1L RNAi/+)* (N – 8), *AFG3L2 KD (;hmlΔ-Gal4>UAS-GFP/+;UAS-AFG3L2 RNAi/+) + NAC* (N- 10), *YME1L KD* *(;hmlΔ-Gal4>UAS-GFP/+;UAS-YME1L RNAi/+)* *+ NAC* (N – 9), *Cat OV* *(;hmlΔ-Gal4>UAS-GFP/UAS-Cat;)* (N-9), *AFG3L2 KD Cat OV* *(;hmlΔ-Gal4>UAS-GFP/UAS-Cat;UAS-AFG3L2 RNAi/+)* (N-8) and *YME1L KD Cat OV* *(;hmlΔ-Gal4>UAS-GFP/UAS-Cat;UAS-YME1L RNAi/+)* (N-14) genotypes.

Images represented are maximum intensity projections of individual slices. White dotted lines are used to mark the lymph gland lobes (pri.- primary lobes, sec.- secondary lobes, tert.-tertiary lobes). Error bars represent SEM. Statistical significance was estimated using Students’ t test with Welch’s correction (ns – nonsignificant, *p<0.05, **p<0.01, ***p<0.001, ****p<0.0001). Scale bars – 100 µm.
